# The Characteristics and Expression Analysis of the Tomato *KWL* Gene Family Under Biotic Stress

**DOI:** 10.3390/genes15121555

**Published:** 2024-11-29

**Authors:** Mei Su, Xuejuan Ru, Yang Chen, Hongjuan Wang, Jia Luo, Hong Wu

**Affiliations:** 1 Chongqing Academy of Agricultural Sciences, Chongqing 401329, Chinaruxuejuan123@163.com (X.R.); lixianchy2008@163.com (Y.C.); hjwang_2005@126.com (H.W.); luojia925@163.com (J.L.); 2 Chongqing Key Laboratory of Adversity Agriculture Research, Chongqing 401329, China; 3 Key Laboratory of Evaluation and Utilization for Special Crops Germplasm Resource in the Southwest Mountains, Ministry of Agriculture and Rural Affairs, Chongqing 401329, China

**Keywords:** *Solanum lycopersicum*, KWL, genome-wide identification, evolutionary relationships, stress response

## Abstract

Background: Tomatoes are renowned for their popularity and nutritional value across the globe, yet their production and quality face significant challenges from various biotic stresses in their growing environments. Kiwellin (KWL) has been implicated in plant disease resistance. However, our comprehension of this gene family in plants is still remarkably insufficient. Methods: We conducted a comprehensive genomic analysis of the *KWL* gene family in tomatoes. The tertiary structures of SlKWLs were predicted by AlphaFold2. EMBOSS was used for codon analysis. RNA-seq and RT-qPCR analysis were performed to explore the expression profile of SlKWLs. Results: Our findings identified 12 distinct SlKWL members distributed across four chromosomes within the tomato genome. By examining their gene structure, conserved motifs, functional domains, and phylogenetic relationships, we elucidated the complex evolutionary relationships and potential functions of these genes. Notably, we identified numerous cis-regulatory elements within the promoter regions of the *SlKWL* genes which are associated with responses to both abiotic and biotic stresses, as well as hormone signaling pathways. This finding strongly implies that SlKWLs are integral to plant growth and adaptation to diverse stress conditions. Furthermore, RNA-seq and RT-qPCR analysis revealed an upregulation of five *SlKWLs* expressed subsequent to *Phytophthora infestans* infection. Particularly, SlKWL2 and SlKWL3 exhibited substantially elevated expression levels, underscoring their active involvement in biotic stress responses. Conclusions: Collectively, these findings advance our comprehension of the *SlKWL* gene family and provide a robust foundation for future investigations into the roles of *SlKWL* genes in tomato stress responses.

## 1. Introduction

Tomato (*Solanum lycopersicum*) is a representative crop species of *Solanaceae* and holds immense economic value as a vital vegetable crop, as well as having high nutritional value and health benefits for humans [1,2,3]. Tomatoes face persistent threats from various stresses in their environment, particularly diseases that jeopardize their growth and reproduction. Such challenges can result in significant declines in both yield and quality [4]. Therefore, it is crucial to enhance the resistance of tomato plants to these biotic stresses to safeguard their productivity and ensure food security.

Late blight, caused by the notorious oomycete *P. infestans*, stands as one of the gravest threats to *Solanaceous* crops, including tomatoes [5]. This disease continues to pose significant challenges to both tomato and potato production worldwide. *P. infestans* is classified within the *Peronosporaceae* family of the phylum Oomycota and has the capacity to infect all parts of the tomato plant, including the stems, leaves, and fruits [6,7]. Following the infection of a susceptible host, local lesions can rapidly spread throughout the plant in just a few days, often leading to its death [8]. 

Kiwellins (KWLs), known for their cysteine-rich nature, form a significant part of the protein structure in kiwifruit (*Actinidia* spp.) [9,10,11], and were first recognized as a new allergenic compound [9,12,13]. Several pathogenesis-related (PR) proteins have been found and analyzed in kiwifruit, including kiwellin, thaumatin-like proteins, actinidain, and kirola [9,11,14]. While the functions of actinidin [14] and thaumatin-like proteins are fairly well established [15,16], knowledge regarding the roles of kirola and kiwellin remains limited [17]. This raises an important question: what accounts for their abundance in kiwifruit, and what is their functional relevance in the plant?

Kiwellins have been identified as plant defense molecules, and their common occurrence in various plant species implies an evolutionarily conserved role in controlling biotic interactions [17,18]. The structural similarity between kiwellins and barwin is quite striking. These barwin and barwin-like proteins fall under the PR4 family of PR proteins, predominantly found in plants but also present in bacteria, algae, and fungi [19]. The barwin domain is multifunctional, capable of binding sugars, cleaving RNA and DNA in the presence of divalent cations, and displaying antifungal properties [20]. Previous study showed that *RRP1* (kiwellin gene) is induced by Pepper golden mosaic virus (PepGMV) in peppers. Its transcript is notably abundant in symptomatic leaves (9 dpi) but decreases in recovered tissues (20 dpi), suggesting a role in viral resistance [21]. Furthermore, the expression levels of two kiwellin homologous genes (*TC197025* and *KiTH-2*) are strongly induced by *P. infestans* in potatoes (*S. tuberosum*) [22,23]. Although kiwellin transcripts are elevated under biotic stress, direct evidence linking kiwellin to disease resistance was limited until the discovery of ZmKWL1 and ZmKWL1-b in maize (*Zea mays*). These two paralogs in maize function redundantly to confer resistance to smut disease [17,24]. ZmKWL1 acts by disabling the effector UmCmu1, preventing salicylic acid biosynthesis through physical interaction, which improves plant disease resistance [17]. Likewise, ZmKWL1-b interacts with UnCmu1 to significantly diminish the pathogen’s impact [24]. A different study found that GhKWL1 boosts plant defense against *Verticillium dahliae* through signaling mediated by ERF [25]. Yet, despite these findings, the roles of kiwellin family members remain poorly defined, particularly due to their lack in Arabidopsis [17].

Plants have evolved a range of mechanisms and intricate signaling networks that allow them to swiftly respond to unpredictable external conditions throughout their long evolutionary history [26]. One of the most crucial aspects of this adaptive capability is the rapid alteration of gene expression in response to challenging biological and abiotic stresses. Therefore, the *KWL* gene family within the tomato genome was identified, and their expression profiles in response to *P. infestans* infection were examined, in our study. This study provides a strong basis for future analysis of potential tomato *KWL* genes linked to biotic stress tolerance.

## 2. Materials and Methods

### 2.1. Detection of *KWL* Gene Family Members and Establishment of a KWL Evolutionary Tree in Tomatoes

The *KWL* gene family was conclusively identified by BLAST searches conducted in the Sol Genomics database (http://solgenomics.net/, accessed on 15 September 2024) [27]. We utilized maize KWL protein sequences identified in MaizeGDB (https://maizegdb.org/, accessed on 12 September 2024) [28] and kiwifruit KWL protein sequences identified in the kiwifruit genome database (https://kiwifruitgenome.org, accessed on 13 September 2024) [29] as query sequences. Then, the CDD search function in NCBI (https://www.ncbi.nlm.nih.gov/Structure/bwrpsb/bwrpsb.cgi, accessed on 18 September 2024) and the InterPro tool (https://www.ebi.ac.uk/interpro/, accessed on 18 September 2024) were applied to validate the kiwellin domain’s presence in the resulting twelve non-redundant KWL protein sequences. The full-length KWL protein sequences of tomato and the other four species (rice, kiwifruit, tobacco, and maize) were subjected to TBtools-II (v2.136) [30]. Subsequently, One Step Build a ML Tree function was used to construct a phylogenetic tree with default parameters. The gene names and accession numbers utilized in this phylogenetic tree are listed in Supplementary File S1.

### 2.2. Investigation into the Physical and Chemical Features of Tomato KWL Proteins

The amino acid number, molecular weight (MW), instability index, aliphatic index, grand average of hydropathicity (GRAVY), and theoretical isoelectric point (pI) of the KWLs were predicted by the Protein Parameter Calc function in TBtools-II (v2.136). 

### 2.3. Subcellular Localization Analyses

Cell-PLoc 2.0 (http://www.csbio.sjtu.edu.cn/bioinf/Cell-PLoc-2/, accessed on 20 September 2024) [31] was applied to determine the subcellular localization of the SlKWLs.

### 2.4. Phylogenetic Relationship, Protein Secondary Structure, Gene Structure, and Motif Analysis of Tomato SlKWLs

The Sol Genomics database (http://solgenomics.net/, accessed on 23 September 2024) was used to download the tomato genome, annotations, and protein sequence files (SL4.0) [27]. MEGA-X [32] was used to create the phylogenetic tree of *SlKWL* genes, and the exon-intron structures were displayed with the Gene Structure Visualization function in TBtools-II (v2.136). The MEME Suite tools (http://meme-suite.org, accessed on 24 September 2024) [33] were used to predict conserved motifs, with the motif parameters manually restricted to 8. TBtools-II (v2.136) was used to illustrate the motifs.

### 2.5. Cis-Acting Elements Analysis of *KWL* Gene Family in Tomatoes

TBtools-II (v2.136) software was used to extract the promoter regions, which are 2000 bp upstream of the start codon, of the *SlKWL* genes. For analysis, the promoter sequences were entered into the PlantCARE online database (https://bioinformatics.psb.ugent.be/webtools/plantcare/html/, accessed on 26 September 2024) [34], and these cis-regulatory elements were drawn by TBtools-II (v2.136).

### 2.6. Prediction of Phosphorylation Sites and N-Glycosylation Sites

The putative phosphorylation sites (Ser/Thr/Tyr) and N-glycosylation sites (tipo Asn-XSer/Thr) were predicted using the NetPhos 3.1 web-based tool (https://services.healthtech.dtu.dk/services/NetPhos-3.1/, accessed on 11 October 2024) [35], and the NetNGlyc 1.0 server (http://www.cbs.dtu.dk/services/NetNGlyc/, accessed on 15 October 2024) [36], respectively. Phosphorylation sites and N-glycosylation sites were draw by IBS (http://ibs.biocuckoo.org/online.php, version 1.0, accessed on 12 October 2024) [37].

### 2.7. Analysis of Chromosomal Position and Gene Duplication

TBtools-II (v2.136) was employed to map the 12 tomato *KWL* genes, identified from the Sol Genomic Database, to their respective chromosomal locations. *SlKWL* gene duplication events were assessed with the one-step MCScanX algorithm in TBtools-II software (v2.136) and confirmed through BLASTP with an E-value limit of 10^−10^. The Advance Circos function in TBtools-II software (v2.136) was employed to visualize the identified duplicated gene pairs.

### 2.8. Tertiary Structure Prediction of SlKWL Proteins

AlphaFold2 (ColabFold v1.5.5) software [38] was used to predict the tertiary structure of SlKWLs. The model with the highest pLDDT values out of the five generated was chosen as the final result. The tertiary structure of SlKWL proteins was visualized with PyMOL 2.5 (https://pymol.org/2/, accessed on 25 March 2024).

### 2.9. Compositional Features of Nucleotide Sequences of *SlKWL* Genes

EMBOSS (https://www.bioinformatics.nl/cgi-bin/emboss/cusp, accessed on 25 December 2024) was used for codon analysis.

### 2.10. Plant Materials, Growth Conditions, and P. infestans Infections

*S. lycopersicum* CLN2037 (resistant cultivar) and LA4084 (susceptible cultivar) plants (two varieties kept in our lab, which were commonly used for resistant and susceptible materials, respectively) were grown in a controlled environment at 25 °C (light/dark, 16/8 h) with a light intensity of 300 µmol·m^−2^s^−1^ and humidity maintained at 60–70%. *P. infestans* “P12103” was cultured in oat medium at 20 °C in the dark for 20 days. The plates covered with mycelium were washed with distilled water until they contained 10^6^ zoospores/mL. After this, the spore suspensions were cooled at 4 °C for 1–2 h to facilitate the release of the zoospores. For each group, 30 tomato seedlings with consistent growth were chosen from both the resistant and susceptible varieties. The prepared spore suspension was applied to tomato plants with six fully expanded true leaves, while the control group was treated with sterile water. Three independent biological replicates were used for each sample, with each group consisting of three seedlings. Plant leaves were gathered at 0, 12, 24, and 48 h post inoculation (hpi). After collection, the leaves were swiftly frozen in liquid nitrogen and stored at −80 °C for future DNA and RNA extraction.

### 2.11. RNA Sequencing (RNA–seq) Analysis

Tomato leaves were used in triplicate to extract total RNA with TRIzol^®^ Reagent, following Invitrogen’s instructions (Thermo Fisher Scientific, Waltham, MA, USA), and DNase I from TaKara (TaKaRa Bio, Inc., Tokyo, Japan) was used to remove genomic DNA. The quality of RNA was evaluated using an Agilent 2100 Bioanalyzer and measured with a NanoDrop ND–2000 from NanoDrop Technologies. RNA-seq transcriptomic libraries were prepared using the TruSeq™ RNA Sample Preparation Kit (Illumina, San Diego, CA, USA), with 1 μg of total RNA as the input. Messenger RNA was isolated using poly-A selection, fragmented, and then used to synthesize double-stranded cDNA with a SuperScript double-stranded cDNA synthesis kit (Thermo Fisher Scientific, Waltham, MA, USA). Libraries were size-selected for 300 bp cDNA fragments after end-repair and phosphorylation, followed by 15 cycles of PCR amplification. Libraries were quantified via TBS380 and sequenced on the Illumina NovaSeq 6000 system. SeqPrep and Sickle, with their default settings, were employed to trim and quality control the raw paired-end reads. Clean reads were aligned to the *S. lycopersicum* reference genome (SL 4.0) [27]. The trend chart was drawn through SRplot (https://www.bioinformatics.com.cn, accessed on 25 October 2024), an online platform for data analysis and visualization.

### 2.12. RNA Extraction and RT-qPCR Analysis

Total RNA from tomato leaves was extracted using the TIANGEN RNAprep Pure kit (TIANGEN Technology Company, Beijing, China). Reverse transcription was performed on 1 μg of RNA to generate cDNA with the FastKing One Step RT-PCR Kit. Quantitative real-time PCR was conducted using FastFire qPCR SYBR Green Master Mix reagent (TIANGEN Technology Company, Beijing, China). NCBI Primer-BLAST (https://www.ncbi.nlm.nih.gov/tools/primer-blast/, accessed on 8 October 2024) was employed to design the specific primers for RT-PCR in this study (Table S1). The tomato actin gene (Solyc03g078400) was used as an internal reference for expression levels. The relative expression of target genes was determined using the 2^−∆∆CT^ method, with each experiment conducted in triplicate.

## 3. Results

### 3.1. Identification of the SlKWL Genes in Tomato

**Figure 1:**
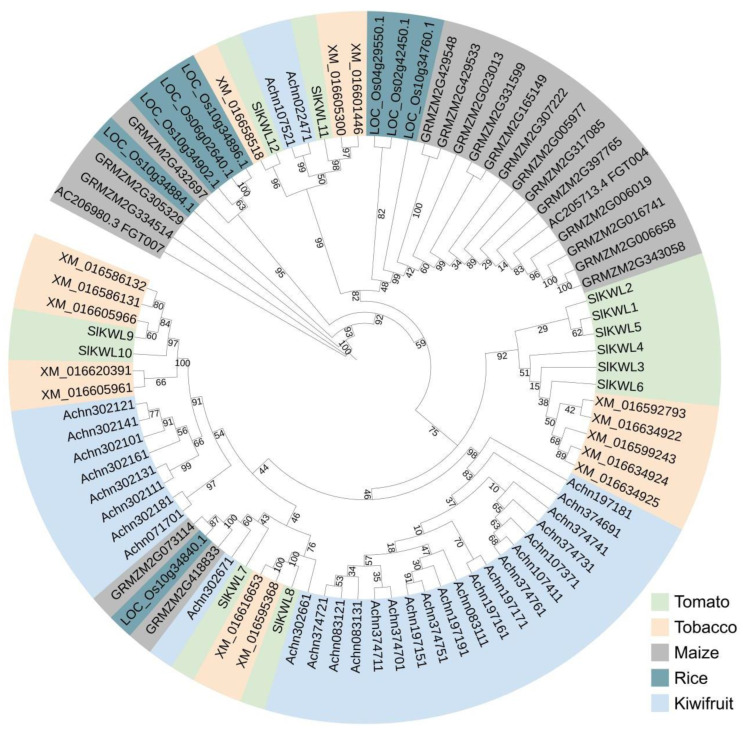
Phylogenetic analysis of SlKWL family proteins and the other homologs. The phylogenetic tree was constructed with a ML method using TBtools-II (v2.136).

To identify KWL proteins, we conducted a query search against the tomato genome database using maize and kiwifruit KWL proteins as reference controls. Further validation of the conserved structural domain was conducted using NCBI-CDD and InterPro. In total, 12 KWL family members with complete kiwellin-like domains were identified in the tomato genome (Supplementary File S1). These proteins were designated, in accordance with their chromosomal locations, as SlKWL1 to SlKWL12 (Supplementary File S1). To further investigate the evolutionary relationships among KWL homologs from various plant species, we also identified 53 KWL proteins from four additional species, including dicotyledonous plants such as tobacco and kiwifruit, as well as monocotyledonous plants like rice and maize (Supplementary File S1). We then built a multispecies phylogenetic tree employing the maximum likelihood (ML) approach. (Figure 1). The analysis of evolutionary relationships shows that tomato KWL proteins are closely linked to those in tobacco, suggesting potential common functions or evolutionary paths.

### 3.2. Analysis of the Motif, Domain, and Gene Structure of Tomato SlKWL Family Members

**Figure 2:**
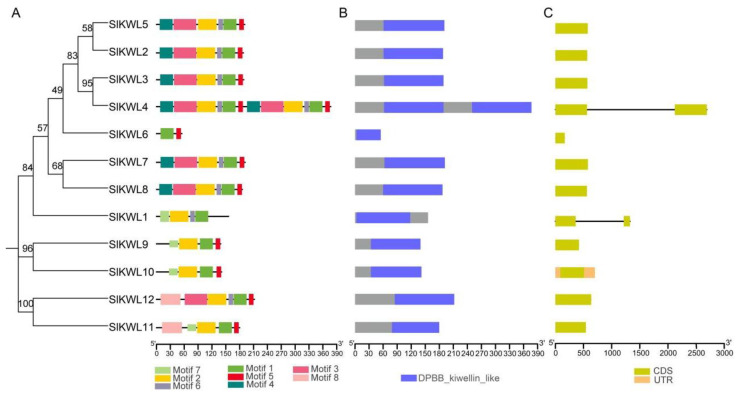
The protein motif, conserved protein domains, and gene structure of SlKWLs based on their evolutionary relationship. (**A**) Evolutionary analysis of SlKWLs, and conserved motif visualization of SlKWLs; (**B**) conserved domain visualization; (**C**) gene structure visualization. CDS: coding sequences; UTR: untranslated regions.

We established a phylogenetic tree of SlKWLs, followed by a detailed analysis of gene motifs, conserved domains, and gene structure information (Figure 2). To explore the diversity within the SlKWL family, we analyzed 12 SlKWL proteins for conserved motifs using the MEME tool, with the identification of 8 conserved motifs (Figure 2A). Motifs were discovered to be extensively present in all SlKWL proteins. Overall, phylogenetically related members tend to have similar motif compositions. According to the results of motif analysis, all SlKWL proteins contain at least four motifs, except SlKWL6, which uniquely features only two. Motif 8 is present exclusively in two SlKWLs (SlKWL11, and SlKWL12), while motif 7 appears only in four SlKWLs (SlKWL1, SlKWL9, SlKWL10, and SlKWL12). In addition, motif 2 is observed in all SlKWLs except SlKWL6. Motifs 1–6 are found in six members, while motifs 1–3 and 5–6 are present in seven members, and motifs 1, 2, and 5 are detected in ten members; these results indicated a high level of conservation across the *SlKWL* gene family. The motif evaluation further suggests that the quantity and distribution of KWL proteins within the same phylogenetic branch exhibit greater similarity. For conserved protein domain analysis using InterPro, we found that 11 SlKWL proteins possess a single kiwellin domain, with SlKWL4 containing two kiwellin domains (Figure 2B). We also examined the gene structure of the *SlKWL* genes, revealing that most *SlKWLs* lack introns (Figure 2C). Among the 12 *SlKWLs* analyzed, only two (*SlKWL1* and *SlKWL4*) contain a single intron, suggesting a distinct evolutionary pattern for these genes. Notably, *SlKWL4* is the longest gene in the family, spanning 2692 bp, setting it apart from the others.

### 3.3. Analysis of Physicochemical Properties of *SlKWL* Gene Family

The *SlKWL* genes’ physicochemical properties are presented in Table 1, and their coding sequences (CDS) span from 168 to 1134 nucleotides. The resulting amino acid lengths for the tomato KWL family vary between 55 amino acids and 377 amino acids. These proteins exhibit molecular weights that span from 6151.61 Da to 40,364.99 Da. Among these proteins, SlKWL6 has the lowest relative molecular mass at 6151.61 Da, while SlKWL4 has the greatest relative molecular weight of 40,364.99 Da. Upon predicting the physicochemical properties, we observed that the isoelectric points (pI) of the 12 SlKWL proteins span from 4.78 to 8.73. Five proteins—SlKWL1, SlKWL7, SlKWL9, SlKWL10, and SlKWL12—are classified as basic proteins (pI > 7), while the remaining seven proteins are acidic proteins (pI < 7). The highest aliphatic index of 87.63 was recorded for SlKWL9, whereas SlKWL6 exhibited the lowest aliphatic index at 53.09. Additionally, the GRAVY (Grand Average of Hydropathicity) values for all 12 SlKWL proteins are below zero, indicating that these proteins are hydrophilic. The instability index ranges from 35.26 to 61.61, with six SlKWLs showing low instability indices (<40), suggesting that approximately 50% of SlKWLs are theoretically stable. Predictions for subcellular localization indicate that most SlKWL proteins are predominantly localized in the nucleus and cell membrane (Table 1).

### 3.4. Analysis of Cis-Acting Elements in *SlKWL* Genes

**Figure 3:**
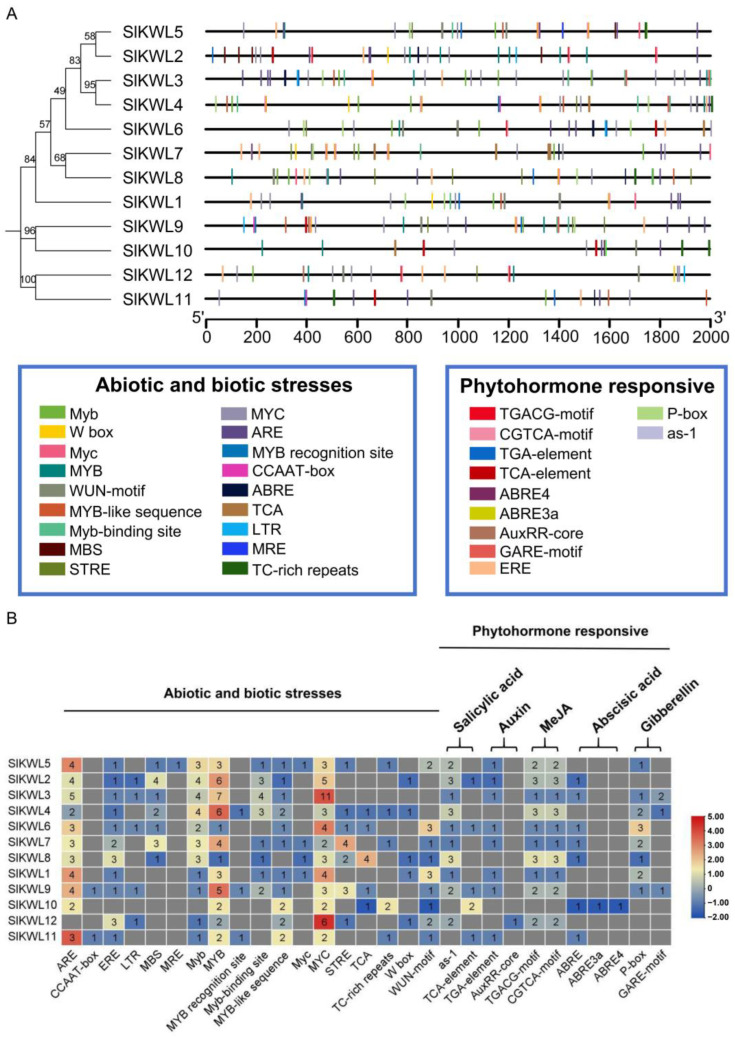
Cis-acting elements in *SlKWL* genes. (**A**) Cis-acting elements of *SlKWL* promoters were predicted with PlantCARE. (**B**) Numbers in boxes are the numbers of each element in the promoter region. The colors within the boxes denote the numerical value. MeJA: Methyl jasmonate.

A 2000 bp region upstream of the 5′ UTR was extracted from tomato genomic data to serve as the promoter sequence of *SlKWLs* (Supplementary File S2). These promoter regions’ cis-acting elements were discovered on the PlantCare website. Our study uncovered a diverse array of cis-acting elements, especially those linked to responses to biotic and abiotic stress, along with elements responsive to hormones. (Figure 3, Table S2). This indicates that *SlKWLs* are likely involved in responses to both biotic and abiotic stresses, in addition to their hormonal responsiveness (Table S2, Figure 3). Notably, the phytohormone response elements include a greater number of methyl jasmonate response elements (TGACG-motif and CGTCA-motif), followed by those responsive to salicylic acid (as-1 and TCA-element). Furthermore, several gibberellin, auxin, and abscisic acid response elements were also identified, highlighting the multifaceted regulatory roles *SlKWLs* may play in plant stress responses and hormonal signaling.

### 3.5. Chromosomal Position, Gene Duplication, and Microsynteny Analysis of *SlKWL* Genes

**Figure 4:**
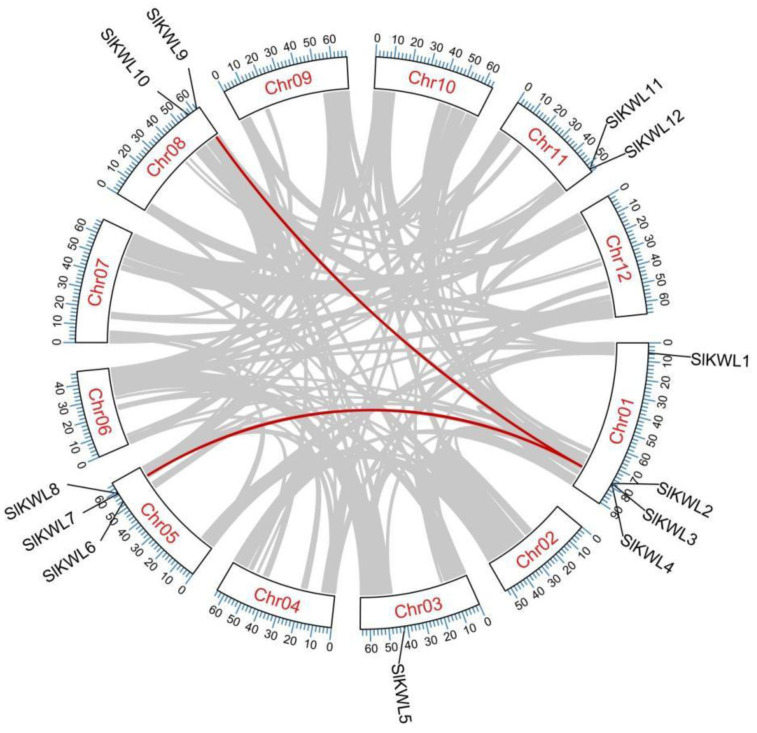
The positions on chromosomes and synteny relationships of *SlKWL* gene pairs in tomato plants are illustrated. The syntenic genes are marked with colored lines, and the gray lines indicate the collinear blocks of plant genomes. Chr refers to chromosome.

In addition, we conducted a chromosomal localization analysis of *SlKWLs*, revealing that there is an uneven distribution of these genes on five chromosomes (Figure 4). Specifically, *SlKWL5* is positioned on chromosome 3, while *SlKWL9* and *SlKWL10* are located on chromosome 8. Likewise, *SlKWL11* and *SlKWL12* are situated on chromosome 11, with *SlKWL6-8* being found on chromosome 5, and *SlKWL1-4* being located on chromosome 1. To examine potential gene duplication within the *SlKWL* gene family, a collinearity analysis of the tomato genome was carried out using the One Step MCScanX feature in TBtools-II. This analysis identified only two syntenic gene pairs within the *SlKWL* gene family, and the absence of genes within a 100 kb region on the same chromosome meant that no tandem duplication events were detected. (Figure 4). These findings indicate that some of the tomato *KWL* genes may have originated from gene duplication events, providing insight into their evolutionary history.

### 3.6. Phosphorylation Sites and N-Glycosylation Sites Analysis of *SlKWL* Gene Family

**Figure 5:**
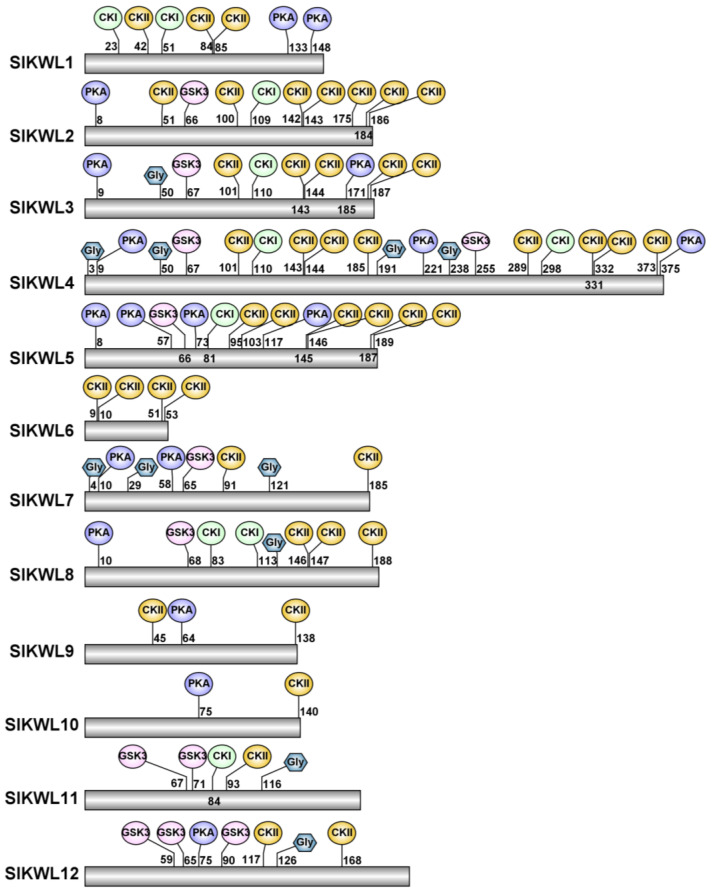
Analysis of phosphorylation and N-glycosylation sites in SlKWL proteins. Phosphorylation sites are depicted by ellipses, while hexagons indicate N-glycosylation sites.

Phosphorylation and N-glycosylation, as post-translational modifications, are essential in regulating stress-related proteins in plants under stress. [39,40]. For identifying phosphorylation and N-glycosylation sites in tomato KWL proteins, we employed the NetPhos 3.1 and NetNGlyc 1.0 online resources. Our analysis revealed a significant number of phosphorylation sites across the tomato KWL proteins, with the majority being targeted by CKI and CKII, followed by sites targeted by PKA and GSK3. Additionally, we identified several N-glycosylation sites present in six SlKWL proteins: SlKWL3, SlKWL4, SlKWL7, SlKWL8, SlKWL11, and SlKWL12 (Figure 5). This information underscores the potential regulatory mechanisms of SlKWL proteins in mediating plant stress responses.

### 3.7. Prediction of the Tertiary Structure of SlKWL Proteins

**Figure 6:**
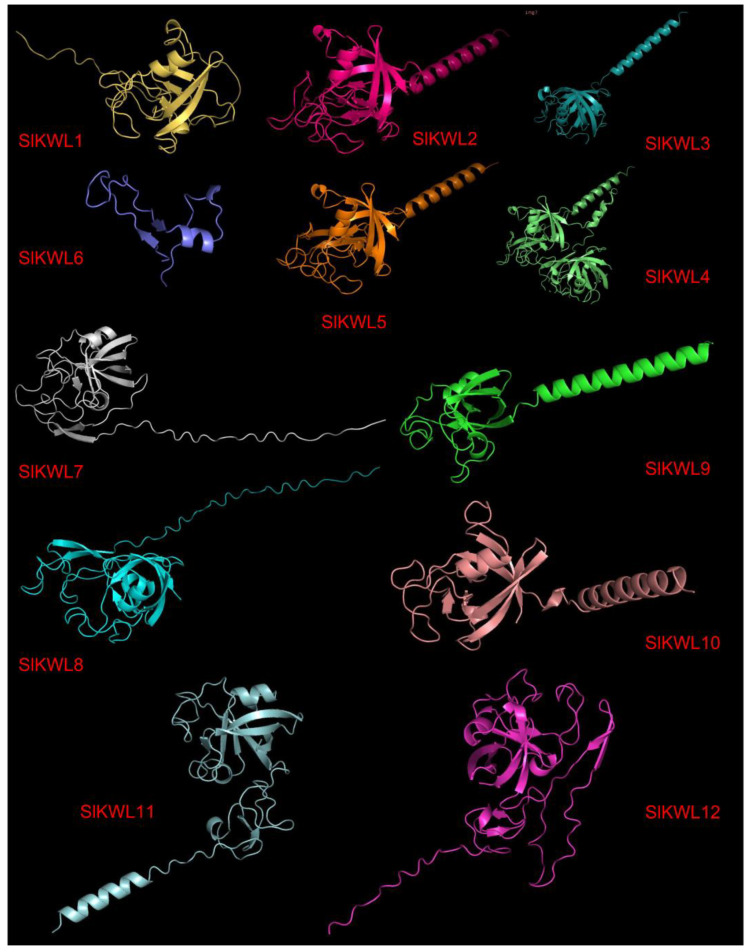
Prediction of the tertiary structures of tomato SlKWL proteins.

The tertiary structures of SlKWL proteins were predicted by AlphaFold2 software (Figure 6). The results indicated that the tertiary structures of the protein combinations (SlKWL2 and SlKWL5, SlKWL3 and SlKWL4, SlKWL7 and SlKWL8, SlKWL9 and SlKWL10, SlKWL11 and SlKWL12) exhibit notable similarities, suggesting that the tertiary structures of KWL proteins within the same phylogenetic branch exhibit greater similarity. These outcomes offer a solid groundwork for future exploration of the functions of KWL proteins.

### 3.8. Codon Analysis of *SlKWL* Genes

The GC content of the *SlKWL* CDSs ranged from 35.21% (*SlKWL10*) to 45.01% (*SlKWL1*), averaging 41.69% (Table S5). The GC content at the first, second, and third codon positions in the *SlKWL* CDSs was also evaluated (Table S5). At the first codon position, GC content ranged from 38.03% (*SlKWL10*) to 55.36% (*SlKWL6*), averaging 45.73%. At the second position, it ranged from 37.86% (*SlKWL10*) to 52.11% (*SlKWL12*), averaging 45.64%. At the third position, it ranged from 25.71% (*SlKWL9*) to 41.18% (*SlKWL7*), averaging 33.70%.

### 3.9. Expression Profiles of the *SlKWL* Genes Based on RNA-seq and RT-qPCR Analysis

**Figure 7:**
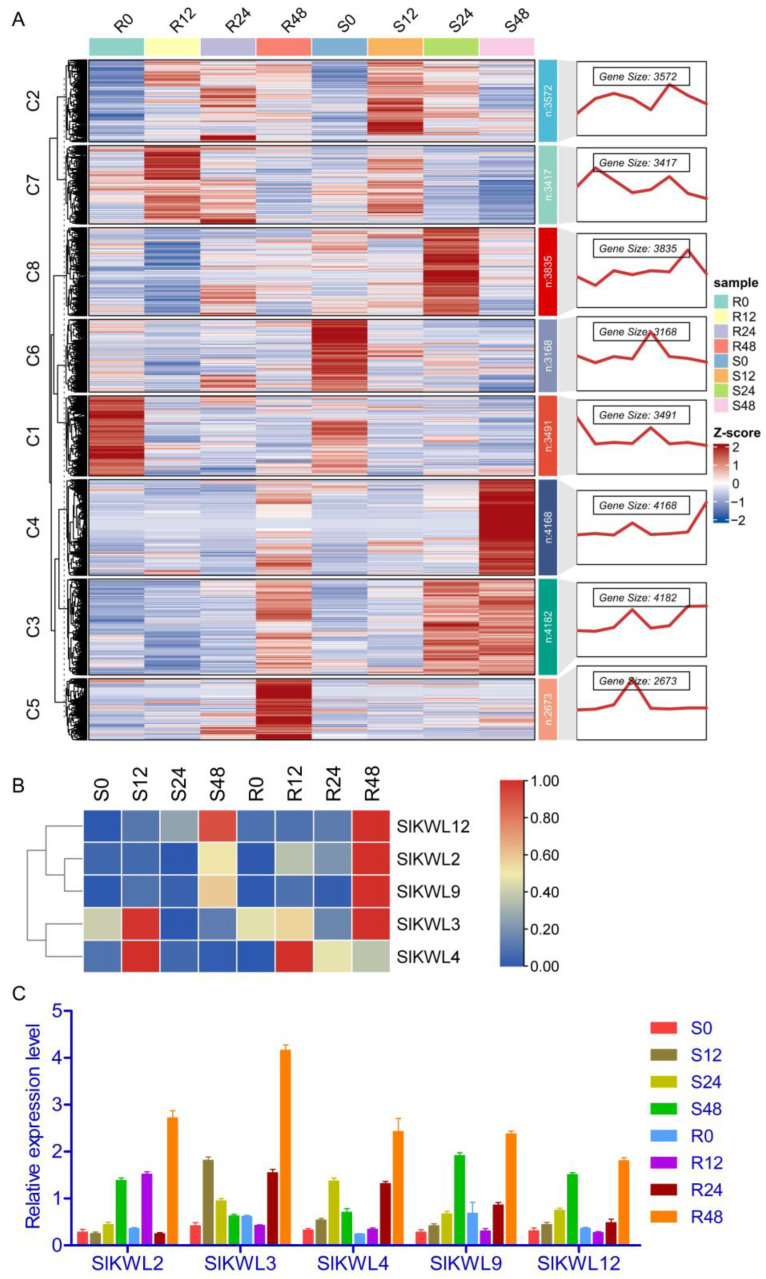
Trend chart of RNA-seq data (**A**) and Expression level of *SlKWL* genes under *P. infestans* infection based on RNA-seq (**B**) and RT-qPCR (**C**) data. R and S represent resistant cultivar and susceptible cultivar, respectively.

To explore the role of the *SlKWLs* under biotic stress, RNA-seq and RT-qPCR analysis were conducted to examine the expression patterns of *SlKWLs* in tomato leaves infected with *P. infestans* at various time points: 0, 12, 24, and 48 h post-infection. A total of 1.2 billion clean paired-end reads were obtained from the RNA-seq dataset for 24 samples, with a mapping rate ranging from 74.2% to 94.3% to the *S. lycopersicum* genome (Tables S3 and S4). Gene expression trend analysis showed that genes belong to cluster 5 (C5), including *SlKWL2* and *SlKWL3*, were induced more strongly in the resistant variety than that in the susceptible variety (Figure 7A, Table S6); these genes were suggested to be potentially involved in plant resistance. Transcriptome data showed that no expression was detected in seven *SlKWL* genes at the four time points, while the other five *SlKWL* genes exhibited up-regulation in response to *P. infestans* infection (Figure 7A, Table S5). RNA-seq and RT-qPCR analysis results indicated that the expression levels of *SlKWL2* and *SlKWL3* were induced more strongly in the resistant variety than in the susceptible variety (Figure 7, Table S5), hinting that these two genes may participate in the tomato’s reaction to *P. infestans* infection, highlighting their potential significance in biotic stress resistance.

## 4. Discussion

Late blight, caused by *P. infestans*, commonly affects tomatoes [5]. The primary targets of late blight are the roots, stems, leaves, and other plant components, resulting in major yield reductions and poor fruit quality [6,7]. Due to the constraints of chemical control, it is important to investigate the genes and molecular mechanisms linked to *P. infestans* resistance in tomatoes. To defend against pathogens, plants utilize several strategies, one of which is efficient gene-for-gene interaction involving host resistance genes and pathogen avirulence genes [41]. Although previous studies have identified three *KWL* genes from maize and cotton involved in disease resistance, the roles of *KWL* family members in tomatoes remain largely unexplored [17,24,25]. Consequently, the present study aims to identify the tomato *KWL* gene family and investigate their potential contributions to resistance against late blight. 

Various plant groups, including dicots and monocots, have been identified to contain KWL proteins [17]. Nonetheless, a comprehensive identification and analysis of the *KWL* gene family across various plant species has not yet been performed. In this study, we discovered and thoroughly examined 12 unique *KWL* genes across the entire tomato genome, naming them *SlKWL1* to *SlKWL12* according to their positions on the chromosomes (Supplementary File S1). We conducted a phylogenetic analysis to investigate the evolutionary connections between *KWL* homologs from various plant species (Figure 1). This examination indicated a strong connection between KWL proteins in tomatoes and those in tobacco, pointing to potential functional parallels and evolutionary routes shared by these species.

Genes that have comparable structures and preserved motifs frequently indicate similar functions in the analysis of plant evolutionary relationships [42]. Our findings revealed that phylogenetically related members of the *SlKWL* gene family possess analogous motifs and conserved domain compositions (Figure 2), indicating that they might have come from a shared evolutionary ancestor and developed with comparable functional roles. Additionally, the examination of exon-intron structures is crucial, as conserved patterns among homologous gene families can shed light on their evolutionary developments [43,44]. In our analysis, ten of the *SlKWL* genes were found to lack introns (Figure 2), indicating that intron loss likely occurred during the evolution of this gene family. The loss of introns can facilitate accelerated evolution through duplication processes [44], further emphasizing the dynamic nature of the *SlKWL* gene family.

Characterizing the physicochemical properties of proteins from various gene families is critical for identifying the functions and attributes of the proteins [45]. Our results showed that most members of the *KWL* gene family, which exhibit high homologous conservation in their amino acid coding sequences, possess similar values for the protein Instability Index and Aliphatic Index (Table 1). This consistency in physicochemical properties underscores the functional similarities between the SlKWL proteins and supports their involvement in similar biological processes.

Within promoter sequences, cis-acting elements play a critical role in plant regulatory networks, providing insights into transcriptional regulation and the functions of related genes [46,47]. In this study, by predicting the *SlKWL* promoter sequences, we identified 375 cis-acting elements predominantly associated with stress responses and hormone signaling (Figure 3, Table S2). These hormone-responsive elements are well-documented as key players in the plant’s reaction to both abiotic and biotic stresses [48,49]. Therefore, the study of these cis-acting elements implies that *SlKWL* genes are likely part of the tomato’s diverse responses to various stress conditions.

In eukaryotes, gene duplications, including segmental and tandem duplications as well as inversion events, are typical processes that lead to the growth of gene family members and enhance genome complexity [50,51]. In our analysis of the *SlKWL* gene family, we identified two syntenic gene pairs; however, we did not observe any tandem duplication events, because no genes were positioned within the same 100 kb region on the same chromosome (Figure 4). The evidence suggests that segmental duplications could have played a key role in the evolutionary process of the *SlKWL* gene family in tomatoes. The lack of tandem duplications further emphasizes the importance of segmental duplications in the evolutionary history of this gene family.

In general, post-translational regulation is essential in the response to environmental stresses, with phosphorylation and N-glycosylation being common methods of such regulation [39,40]. In our study, we identified numerous phosphorylation sites and several N-glycosylation sites in tomato KWL proteins (Figure 5). These findings suggest that the functions of SlKWL proteins may be modulated by post-translational modifications, highlighting their potential significance in the plant’s adaptive responses to stress conditions. This regulatory mechanism can enhance the functional diversity and adaptability of these proteins in various physiological contexts. 

The prediction results of tertiary structures suggest that the tertiary structures of SlKWL proteins within the same phylogenetic branch exhibit greater similarity (Figure 6). Insights into synthetic biology may be gained from the GC content of the SlKWL CDSs and the GC content at the first, second, and third codon positions (Table S5).

Pathogenic infections lead to a notable upregulation of *SlKWL* gene expression in various plants, including pepper, potato, cotton, and maize [17,21,22,23,25]. Consistent with these previous studies, our analysis of the *SlKWLs*’ expression profile demonstrated that *SlKWL* transcripts are induced by *P. infestans* infection in both resistant and susceptible tomato cultivars (Figure 7, Table S7). Notably, *SlKWL2* and *SlKWL3* exhibited remarkable induction following *P. infestans* infection, with even stronger expression observed in the resistant cultivar “CLN2037” compared to the susceptible cultivar “LA4084.” These results suggest that *SlKWL2* and *SlKWL3* may play roles in conferring resistance to late blight. Overall, these findings enhance our understanding of the *SlKWL* gene family and establish a solid basis for further exploration of the functional roles of *SlKWL* genes in tomatoes.

## Figures and Tables

**Table 1:** Gene structure and protein properties of SlKWLs in tomato.

ID	CDS (bp)	Protein Length (AA)	MW (Da)	pI	Instability Index	Aliphatic Index	GRAVY	Subcellular Localization
SlKWL1	471	156	17,408.39	7.54	61.61	57.5	−0.747	Nucleus
SlKWL2	567	188	20,243.51	4.62	36.26	63.78	−0.469	Nucleus
SlKWL3	570	189	20,234.53	4.46	38.21	63.44	−0.447	Cell wall. Nucleus.
SlKWL4	1134	377	40,364.99	4.65	35.26	65.94	−0.446	Cell membrane. Nucleus.
SlKWL5	576	191	20,683.06	4.78	46.85	60.26	−0.541	Cell membrane. Cell wall.
SlKWL6	168	55	6151.61	3.66	36.11	53.09	−0.729	Cell membrane. Nucleus.
SlKWL7	561	186	20,062.98	8.71	50.46	64.46	−0.409	Nucleus.
SlKWL8	579	192	21,450.36	6.09	44.59	76.61	−0.466	Nucleus.
SlKWL9	420	139	15,455.8	7.63	48.31	87.63	−0.133	Cell membrane. Chloroplast. Cytoplasm.
SlKWL10	426	141	15,884.03	8.32	42.52	74.68	−0.343	Cell membrane.
SlKWL11	543	180	19,003.34	5.15	37.63	70.89	−0.293	Cell membrane. Nucleus.
SlKWL12	639	212	22,372.29	7.91	38.81	70.8	−0.197	Cell membrane. Cell wall.

CDS: coding sequence size; MW: molecular weight (Da); pI: theoretical isoelectric point; GRAVY: grand average of hydropathicity.

## Data Availability

The original contributions presented in the study are included in the article/Supplementary Materials, further inquiries can be directed to the corresponding author.

## Supplementary Materials

The following supporting information can be downloaded at: https://www.mdpi.com/article/10.3390/genes15121555/s1, Table S1: Primers used in this study. Table S2: Cis-Regulatory elements in *SlKWL* genes. Table S3: RNA-seq quanlity. Table S4: Gene expression (FPKM) of transcriptomic data. Table S5: Codon analysis results. Table S6: Gene to cluster. Table S7: The expression level of SlKWLs obtain from RNA-seq and RT-qPCR data. Supplementary File S1: KWL protein sequences. Supplementary File S2 promoter sequences.
