# Supplementary material for: The Characteristics and Expression Analysis of the Tomato KWL Gene Family Under Biotic Stress
Source: Genes (Basel). 2024 Nov 29;15(12):1555. doi: 10.3390/genes15121555 (PMC11675693; doi:10.3390/genes15121555)
Supplement: Supplementary file 1 [file genes-15-01555-s001.zip › Supplementary file S1 KWL protein sequences.pdf]

>Solyc08g080950.1 SlKWL9
MKETILGYVVALLILICMVFNNILIVEAQKHNGHMKAILTINDFSKGGDGGGPS
ECSAKYYHNSVPIVALSTRWYNKGKRCFDKITIYANGKSTKAMVVDECDTSR
GCKSNIVDASEAVWKNLGVPKEDWGWLEVHWSD
>Solyc05g048790.1 SlKWL8
MKNIRLCTKSLIVLLILPTICSFSESRLQVCKPNSKIKAKNPPNGHCNIEEDDICC
IKDKIYTTYKCSPQVSSKTKAILTLNSFEKGGDGDGDGPSKCDNKYHFDDTHV
VALSTGWYNGEGRCLRNITINGNGRSVNAMVVDECDSTMGCDEKHDYQPPC
RNNLVIASQGVWRALRVPINEWGELDITWFDE
>Solyc01g010400.1 SlKWL1
MYTTYKCSPPVTDNTKAILTLNSFENGGNGGGPSECDNQYHSDDTPVVALSIG
WYNGGDRCLNYITISANGRSVKAKVVDECDSTMGCDDEHDYQPPCPNNIVD
ASKAVWEALGIPKGDWAKVYRAVRRRRRSAMETTENYRCRRRRSALSTMRE
V
>Solyc03g013300.1 SlKWL5
MKSTVGTSLLVLLVFATLTYCLDARLQACQPSGKIRDIKPPAGQCNPENDSDC
CKKSKMYTTYKCSPPVTRNTKAILTRNSFEKGGDGGDGGAPSECDNQYHSDD
TPVVALSTRWYSGGDTCLNYITMSANGRSVKAKVVDECDSTMGCDDEHDYQ
PPCPNNIVDASKAVWEALGIPKGDWGDYDITWSDA
>Solyc05g042020.1 SlKWL6
MTVVDECDSTMGCDDEHDYQPPCPNNIVDASKEVWEALGIPKGDWGDYDIT
WSDS
>Solyc01g094430.1 SlKWL2
MKSTVGTSLLVLLVFATLTYCLDARLQACQPSGKIRGIKPPPGQCNTENDSDC
YKKGKMYTTYKCSPPVTGNTKAVLTLNSFQKGGDGGGPSECDNQYHSDDTP
VVALSTGWYSGGDRCLNYITISANGRSVKAKVVDECDSTMGCDDEHDYQPP
CSNNIVDASKAVWEALGIPTDDWGDYDITWSDA
>Solyc01g094440.1 SlKWL3
MKNSIMGASLLVLLVFATLTYCLDARLQGCQPSGKIKGIKPPPGQCNPENDSD
CCKQGKMYTTYTCSPPVTGNTKAVLTLNSFQKGGDGGGPSECDNQYHSDDT
PVVALSTGWYSGGDRCLNYITISANGRSVKAKVVDECDSTMGCDDEHDYQP
PCPNNIVDASKAVWESLGIPEGDWGDYDITWSDA
>Solyc01g094450.1 SlKWL4
MKNSSLGKSLLVLLVFAFLTYCLDARLQGCQPSGKIRGIKPPPGQCNPENDSDC
CKQGKLYTTYTCSPPVTGNTKAVLTLNSFQKGGDGGGPSECDNQYHSDDTPV
VALSTGWYSGGDRCLNYITVSANGRSVKAKVVDECDSTMGCDDEHDYQPPC
PNNIVDASKAVWEALGIPEGDWGDYDITWSDMKNSTLGKLIGVLLIFATLTYC
IDARLQACQPSGSIRGIKPPPGQCNPENDSDCCKQGKIYTTYKCSPPVTGNTKA
VLTLNSFQKGGDGGGPSECDNQYHSDDTPVVALSTGWYSGGDRCLNYITISA
NGRSVKAKVVDECDSTMGCDDEHDYQPPCPNNIVDASKAVWKALGIPDGD
WGDYAITWSDA
>Solyc11g069880.1 SlKWL12
MSNLAILSLIFTIFISTSNAISQCNGPCKTWDDCDGQLICINGKCNDDPNVGTK
VCKNSPPSVPSPTPSNTCRPSGTINCNGVHPIYRCSPPVTSSTPAQLTLNNFARG

GDGGGPSSCDGKYHDNNESIVALSTGWFAGRSRCGKMIRIRANNGKTVTAK
VVDECDSTMGCDAEHAFQSPCKNNIVDGSIAVWRALGLNTNLGIVPVTWSM
V
>Solyc11g069870.1 SlKWL11
MTKLALILSLCIIFTTFISSSYSISSCNGPCKSLNDCDGQLICIKGKCNDDPDVGT
SICGGGGSSPSPPSTGSTSGMLTLNDFSEGGDGGGPSECDEKYHDNNERVVAL
TTRWYDNGSRCGKMIRIRANKNGKSVTAKVVDECDTKDGCKNNVVDGSIAV
WRALGLDTNEGRVPITWSMV
>Solyc05g048780.1 SlKWL7
MKMNTSPKVSILIILTLISCAIAQPSSCNPSGKIEGIKPPPGKCKIGYQSECCKPG
KSYTTYKCSPSISGKTKAVLTINSFQKGGDGGGKSECDNKYHSDNTPVVALST
GWFSGEKRCMQNITIYGNGRKTNAMVVDECDSTMGCDHVHDYQPPCANNI
VDASKAVWKALGVPKKQWGELDIFWSD
>Solyc08g150140.1 SlKWL10
MNNYLILFHFVLLLAYSNTFLMVNALNYKPNDRTKAKLTINDFQKGGSGGGP
SECSGKYYDNSIPVVALSTRWYSKGRRCFENINIYANNGMSTQAMVVDECDT
SRGCKNNIVDASEAVWKNLGISKKDPQWGLMDIFWSD
>LOC_Os10g34902.1
MAANVKVLVVLALLQLMSLHAVVHGGDNGGVSAVATGKHEPKPKQGGGGG
GGDGGCHISGFLHGKAGKCNRAHGSDCCVTGRRYPQFRCSPPVSSARPTPATL
TLNSFARGGDGGGRSSCDGRFHPDTAMVVALSSGWLRLDGASRCNRMIRVAA
GNGRSALARVVDECDSVNGCDAEHNFEPPCPNDVVDGSPAVWKALGLDEGV
GEFKVTWSDV
>LOC_Os10g34760
MANAKQLALFAMLVLLLASCAAARRHGKPDPCDGGGGGVDSHLPPGMRRC
SSPAVSEDGTPAVMTVNGFEEGEDGGGPAACDGRYHSDRSLVAALSTGWFAG
GRRCHRGIRITSRQNGRSVVATVVDECDSRHGGCKDDIVDTSAAVWSALGLD
TNVGEVPVTWSDA
>LOC_Os02g42450.1
MTLNDFEAGGDGGDPSECDGKFHKNTERVVALSTGWYANGRRCNKNIRINA
NGRSVLAKVVDECDSLHGCDKEHAYQPPCRPNVVDASQAVWDALRITGEDV
GEYDITWSDA
>LOC_Os04g29550.1
MAMTNCLLALAIAGVVLVSLPGLSRGDGECNPSGAIRSSTTHRCQDCCKAGQ
SYPTYTCSPPTTGSSTDAVMTLNDFDAGGDGGGPSECDEMYHSNTELVVALST
GWYAGGSRCGKSVRINANGRSVLAKVVDECDSQRGCDEEHAYQPPCRPNVV
DASQAVWDALGITGEDVGEYDITWSDA
>LOC_Os06g02640.1
MEGIKLKFQGYRRDRVNFRGHVLAFCLPHYPWRHGISAAGSCHAGGSGYAYS
TETRRGARCPKGSPDCCVVGKRYPRFMWLTAGIGHAPAIPTLHMCFRRNMEL
VVELYSGSGWLCLDDGGGGARCRRCNGRIRVVVTAAVNGRSVVVRVVDEC
DYVNGCCNEDGFAPPCQDNVGGESLTVWKKLGSTRTGANRSFYSSNRCLTNG
LWTAMALSRRSGKHDGILFNLTKSVVLVGGRIGSSWIIGLVKWVYG
>LOC_Os10g34840.1

MAGAMTMSRRRLSHALLLVLAILPNLAALAVAAGGGSGGGGFFPHRSLLQSS
SCQPSGAITGTSGDCNADNGSECCQDGVQYMTYACSPPVAAGGTGTAALLTL
NSFADGGDGGGAPSCTGRFYDDGQLVVALSTGWFDGRSRCEKDVVIRASGG
ASVTAMVVDECDSQRGCDSDHNFEPPCRNNIVDGSPAVWDALGLNKDDGEA
QITWSDA
>LOC_Os10g34884.1
MPNYDQISNKSGNCEKSNDPDCCEDGKAYPQYRCSPPVTAAAGAVLTLNSFE
KGKDGGGPSECCRRGGSPAWRGAGTGSGSRRAAAAGGRWWRRWWTSATPC
TAATASTTTSRRATTTSWTRRRRCGTPSASTRASGWSTSPGPTMAMSELL
>LOC_Os10g34896.1
MASLMQRPAAMPAATSTSTATRRGVPRGAMTAASPASATRGSGARRRCRSAR
TTPAILTLKVFDHGEDDGGVPTSCDMRFHRNTELVVALSSGWLRLGGGRRRC
HRRIRVFAVAGAASGRSSVVARVVDDCDSVNGCREEDGFAPPCRNNAVGGSP
VVWEKLGLNASVGEFEVVWSCLKNAKETLRTSTICHKNEL
>XM_016586131
MKLSATMNMGTYFTLLHFIAFLILIFMACSNTLIVEAQRHRAPDVKKGKFSPA
VSGHKKAILTINDFRKGGGGGDPSECSGKYYDNSIPIVALSTGWYSKGKRCFE
KITIYANGKSTKAVVVDECNTSKGCRNNIVDASEAVWKALGVPKKDWGWLEI
FWSD
>XM_016586132
MACSNTLTVEAQRHKKPDGKEGKFSPAVSGRRKAILTINDFGKGGGGGGPSEC
SGKYYDNSIPIVALSTGWYSKGKRCFEKITIYANGKSTKAMVVDECDTSRGCR
NNIVDASEAVWKALGVPKKDWGWLEIFWSD
>XM_016592793
MKNSSMGTSLLVLLVFATLTYCSDARLQSCQPSGKIRGIKPPPGQCNPENDSDC
CKQGKMYTTYKCSPPVMGNTKAVLTLNSFQKGGDGGGPSECDNQYHSDDTP
VVALSTGWYSGGDRCLNYITISANDKSVKAKVVDECDSTMGCDDEHDYQPP
CPNNIVDASKAVWEALGIPEGDWGEYDITWSDSCQPSGKIRGIKPPPGQCNPE
NDSDCCKQGKMYTTYKCSPPVTGNTKAVLTLNSFQKGGDGGGPSECDNQYH
SDDTPVVALSTGWYSGGDRCLNYITISADGKSVKAKVVDECDSTMGCDDEH
DYQPPCPNNIVDASKAVWEALGIPEGDWGDFDITWSDA
>XM_016595368
MKNFRLGTTSLIVLLVLATLSSSSNARLPTCKPSGKIKAKKPPTGHCNIEDDDIC
CIKGKVYTTYKCSPPVSNKSTKAVLTLDSFEKGGDGYGPSKCDNKYHSDHTH
VVSLSTGWYNGGGRCLSNITISANNGTSVKAMVVDECDSTMGCDEKHDYQP
PCPNNLVIASQAVWRALHVPMNEWGELDITWSDE
>XM_016599243
MKNSSMGTSLLLLLVFATLTYCSDARLHGCQSSGKIRGIKPPPGQCNPENDSD
CCKQGKMYTIYKCSPPVTGNTKAVLTLNSFQKGGDGGGPSECDNQYHSDDTP
VVALSTGWYSGGDRCLNYITVSANGKSVKAKVVDECDSTMGCDDEHDYQPP
CPNNIVDASKAVWEALDIPEGDWGEYDITWSDACQPSGKIRGIKPPPGECNPE
NDSDCCKQGKMYTTYRCSPPVTGNTKAVLTLNSFQKGGDGGGPSECDNQYH
SDDTPVVALSTGWYSGGDRCLNYITVSANGKSVKAKVVDECDSTMGCDDEH
DYQPPCPNNIVDASKAVWEALGIPEGDWGEYDITWSDA

>XM_016601446
MVKLVFLFLYILAIFISSSWPGNAISSCNGPCNSLNDCDGQLICIRGKCNDDPDV
GTNTCKGRNSPSVPSSPTDSTPAQLTLNDFSKGGDGGGPSECDEKYHDNSERV
VALSTRWYAKGSKCGKMIRIMANNGKSVTAKVVDQCDSINDGCANNVVDGS
IAVWRALGLDTDKGRVPITWSMV
>XM_016605300
MAKFVFLSLCIFAIFISSSSRGTAISSCNGPCKSLNNCDGQLICISGKCNDDPDV
GTNTCKGGGSSPTTSTPAQLTLNDFSKGGDGGGPSECDGQYHDNSERVVALST
RWYAKGSKCGKMILIRANNGKSVTAKVVDECDSINDGCANNVVDGSIAVWR
ALGLDTDKGRVPITWSMA
>XM_016605961
MNKGTYFSLVHFVTFLTLISMVCTNTLVEAQKCKPSGKIKGKKPPKDQCSPGD
ECCKEGKLYTTYKCSPPVSVHTKAILTINDFDKGGDGGGPSECSGTYYHNSIP
VVALSTGWYSKGRRCFENITVHANGRSVKAMVVDECDSGEGCDAPHAYQPP
CPNNIVDASEAVWKALGVPKKDWGWLEISWSE
>XM_016605966
MKLISATMSKGINYLSLAHFVAFLILISMACSNTLVVEAQRHRAPDAKEGKFSL
AVSGRTKAILTINDFRKGGEGGGPSECSGKYYDNSIPIVALSTGWYNKGRRCFE
KITIYANGKSTKAMVVDECDTSRGCRNNIVDASEAVWKALGVPKKDWGWLE
IFWSD
>XM_016616653
MKNFRFGTTSLIVLLVLATLSCPTDARLPTCNPSSKIKAKKPPTGHCNIEDDDIC
CIKGKVYTTYKCSPPVSKKSTKAVLTLDSFEKGGDGYGPSKCDNKYHSDHTH
VVALSTGWYNGGGRCLSNITINANNGISVKAMVVDECDSTMGCDEKHDYQP
PCPNNLVIASQAVWRDLHVPMNEWGELDITWSDE
>XM_016620391
MKLIIAKMNKGIYFSLIHFVTFLILFSLVCTNTLVEAQKCKPSGKIKGKKPPKD
QCSPGDECCKEGKFYTTYKCSPPVTGHTKAILTINDFDKGGDGGGPSECSGTY
YHNSIPVVALSTGWYSKGRRCFENITIHANGKSVKAMVVDECDSGEGCDAPH
AYQPPCPNNIVDASEAVWKALGVPKKDWGWLDISWSE
>XM_016634922
MKNSRGGTSLLVLLVFATLTYCSDARLQACQPSEKIRGIKPPPGQCNPENDSDC
CKQGKMYTTYKCSPPVTGNTKAVLTLNSFQKGGDGGGPSECDNQYHSDDTP
VVALSTGWYSGGDRCLNYITVSANGKSVKAKVVDECDSTMGCDDEHDYQPP
CPNNIVDASKAVWEALGIPEGDWGDYDITWSDVCHPSGKIKGIKPPPGQCNPE
NDSDCCKQGKMYTTYKCSPPVTGNTKAVLTLNSFQKGGDGGGPSECDNQYH
SDDTPVVALSTGWYSGGDRCLNYITISANGKSVKAKVVDECDSTMGCDDEH
DYQPPCPNNIVDASKAVWEALDIPEGDWGDYDITWSDA
>XM_016634924
MKNSSMGTSLLLLLVFAPLTYSSDARLQVCQPSGKIRGTKPPPGQCNPENDSD
CCKQGKMYTTYKCSPPVTGNTKAVLTLNSFQKGGDGGGPSECDNQYHSDDT
PVVALSTGWYSGGDRCLNYITISANGKSVKAKVVDECDSTMGCDDEHDYQP
PCPNNIIDASKAVWEALGIPEGDWGDYDITWSDGCQPSGKIKGIKPPPGQCNP
ENDSDCCKQGKMYTTYKCSPPVTGNTKAVLTLNSFQKGGDGGGPSECDNQY

HSDDTPVVALSTGWYSGGDRCLNYITVSANGKSVKAKVVDECDSTMGCDDE
HDYQPPCPNNIVDASKAVWEALDIPEGDWGEYDITWSEA
>XM_016634925
MMNSSSLLVLLIFATFTYCLDARLQVCQPSGKIIGIKPPPGQCNPENDSDCCKQ
GKMYTTYKCSPPVTGNTKAVLTLNSFQKGGDGGGPSECDNQYHSDDTPVVA
LSTGWYSGGDRCLNYITVSANGKSVKAKVVDECDSTMGCDDEHDYQPPCPN
NIVDASKAVWEALDIPEGDWGDYDITWSDA
>XM_016658518
MATNMIIKLIFLSLCIFTIFISSSSAISSCNGPCQTLDDCDGQLICIRGKCNDDPD
VGTNICNNSPPDPNNCQPSGQVNCHGTHPIYRCSPPVTGSTSAKLTLNDFSEG
GDGGGPSKCDDQYHDNNERIVALSTGWYAGGSRCGKMIRIRANNGKSVTAK
VVDGCDSTTGCDAEHGYQRPCKYNIVDASVAVWRDLGLDTDTGVVPITWSV
A
>Achn022471
MAQLSLLVLTLFLTLISLPPPGASISSCNGPCRDLNDCDGQLICIEGKCNDDPEV
GTHICRGTTPSPQPGGCKPSGTLTCRGKSHPTYDCSPPVTSSTPAKLTNNDFSE
GGDGGGPSECDESYHSNNERIVALSTGWYNGGSRCGKMIRITASNGKSVSA
KVVDKCDSRHGCDKEHAGQPPCRNNIVDGSNAVWSALGLDKNVGVVDITW
SMA
>Achn071701
MTLKLRLADQVVELRVKSPQTNARWSSTPTVASKENSTQHIHVHHQYRHTKA
VLTLSSFSNAIDGGGYPIGRNDKPHFDDTPVVALSTGWYNKGERCLDNITIIAN
GRSVNALVVDKCDSTRGCDEDHDFKPPCGNNVVNVSKEVWKVLGVPRNEW
GELDITWSGR
>Achn083111
MAVACRNSITSTTPMTHMLWHYQLDGTTKEGASMTYIIIRANGRSVRDKVMD
ECDSTMAVLTLNSFQKGGDGGGPSECDNQYHSDDTPVVALSTGWYNKGRRC
LNDIIISANGRSVRAKVVDECDSTMGCDGDHDYQPPCNNNIVDASKAVWEAL
GVSRDNWGEMDITWSDA
>Achn083121
MKTFNLGTSLLILLILIVTHCMETEAQVCRPSGNIRGRKPPPGECNKENDSDCC
AEGKLYPIYRCSPTVFGNTKAVLTLNSFQNGGDGGGPSECDNQYHSDDTPVV
ALSTGWYSKGRRCLNDIIINANGRSVRAKVVDECDSTMGCDGDHDYQPPCN
NNIVDASKAVWEALGVSRDNWGEMDITWSDA
>Achn083131
MKTFNLGTSLLILLILLVTHCIETEAQVCRPSGNIRGRKPPPGECNKENDSDCC
AEDKLYPIYRCSPTVSSNTKAVLTLNSFQKGGDGGGPSECDNQYHSDDTPVVA
LSTGWYNKVFSTFFTVPEPYAYGDFFEIPVLPRVSVMVIRLCIGQYCIVSDDILT
HTHRGERETMKTFNLGTSLLILLILLVTYCMETEAQVCRPSGNIRGRKPPPGEC
NKENDSDCCAEGKLYPIYRCSPTVSGNTKAVLTLNSFQKGGDGGGPSECDNQ
YHSDYTPVVALSTGWYNKGRRCLNDIIISANGRSVRAKVVDECDSTMGCDGD
HDYQPPCNNNIVDASKAVWEALGVSRDNWGEMDITWSDA
>Achn107371
MKTFTLGTSLLILVIVLVTYCLEAEAQVCRPSGNIRGRKPPPGECNQENNSDCC

VEGKLYPIFRCSPTVSGNTKAVLTLNSFQAGGDGGGPSECDNQYHSDDTPVVA
LSTGWYGKGRRCLNDIIISANGRSVRAKVVDECDSTMGCDGDHDYQPPCNN
NIVDASKAVWEALGVPHDQWGELDITWSDA
>Achn107411
MKTFTLGTSLLILVIVLVTYCLEAEAQVCRPSGNIRGRKPPPGECNQENNSDCC
VEGKLYPIYRCSPTVSSNTKAVLTLNSFQAGGDGGGPSE
>Achn107521
MAKFTLLLLSLFLTLITLPPPGAAISSCNGPCQDLNDCDGQLICIKGKCNDDPQ
VGTHICRGGTTPSPQLVGCNPSGTLTCQGQSYPTYDCSPPVKSSTPAKLTNNDF
SKGGDGGGPSECDESYHSNDELIVALSTGWYNGGSRCGKMIQITASNGKRVA
AKVVDECDSRNGCDEEHAGQPPCPNNIVDGSDAVWRALGLDKNVGVVDITW
SMA
>Achn197151
MDESAENSENSLPNTFRAVILAAAELAWDLIVVGVCDGPMLAKKQTVLLVEP
YSYSYTEATIDPLSNKIPSTHGIRHITRQRILTREGKLYPIYRCSPTISSNTKAVLT
LNSFQEGGDGGGPSACDNQYHSDDTPVVALSTGWYNKGRRCLNDIIISANG
RSVRAKVVDECDSTMGCDKDHDYQPPAITISLMPQKTCGRPWECLVTTGEK
WILPGLMLKFFQLLMFR
>Achn197161
MKTFNLGGSLLLLLILLVTHCHEIEAQVCRPSGNIRGRKPPPRECNQENDSDCC
VERKLYPIYRCSPAVSGNTKAVLTLNSFQKGGDGGGPSECNNQYHSNNTPVVA
LSTGWYNKGRRCLNDIIISANGRSVRAKVVNECDSTMGCDGDHDYQPPCNN
NIVDASKTVWEALDVSPDNWEKWISLGPTLKYFSYTCSIKRLFAP
>Achn197171
MNSSTDEGKQNFCVEEKLYPIYRCSPAVFNNTKVVLTLNSFQKGGDGGGSSEC
DNQYHLDDTPYVALSTRRYYKERRFLNDIIISANGRSVRAKVMNECDSTMGC
DEDHDYQLSFNNNIVDASKALIFFTSQNNLISVHPMGRPTPNHSMDDHGIVKC
TDVRQLFRVILRRYLLSIVSKRVEMVVARWNVITSITLMTHQLWHYQPDGME
KEAGASTTSSLVLMGKALRAKVVDECDSTMTLKFVAILGEENLLMENATTRM
APIVVWKENSTQFTDVHQQFLITLRRFLLSTAFKRVEMVVAHQNAITNTTLMT
LQLWHYQPDGTTKEEDASMT
>Achn197181
MNTFSFGATLLLILLIVIGAHCLEAEAQVCRPTGSIRGRKPPPGKCNQENDSDCC
VEGKLYPIYKCSPIVSGNTKAVLTLNSFQKGGDGGGPSECDNQYHSDDTPVVA
LSTGWYNKGRRCLNDIIISANGRSVRAKVVDECDSTMGCDGDHDYQPPCAN
NIVDASKAVWKALGVSRDNWGEMDITWSDA
>Achn197191
MVVAHRSAKTSTILTKHYQPDGMAKEEGVALSTRWYNKGRRCLNNIIISANG
RSVKAKGVDECDSTMGCDGDRDYQSPCNNNTIEASKAVCKALGVPHDQWG
ELDITRSDA
>Achn302101
MKRLCSKTSALLLVTIFLIACFDVEAQSCRPSGKVRGKKPPPNQCNQENDSDC
CVDGHMYTTYKCSPAVSGHTRATLTINSFEAGGDGGGPSECDGKYHFDDLPIV
ALSTGWYNGGNRCFNNITISANGRSVVAMVVDECDSTMGCDKVHDYQPPCP

NNIVDASKAVWKALGVPEDNWGDLDITWSEA
>Achn302111
MRTTYACSPAVSSQTKATLTINSFQAGGDGGGPSERDGKYHSYHLPIVVLSTG
WYNRGRQCFDNITISANGRSVAAMVVNECDSTMGCDQDHEYQTPCDNSIVD
ASKSVWLSASLKITGVAWI
>Achn302121
MKRLCSKTSALLLVTIFLIACFDVEAQSCRPSGKIRGKKPPPNQCNQENDSDCC
VEGHMYTTYKCSPAVSGHTRATLTINSFEAGGDGGGPSECDGKYHSDDLPIVA
LSTGWYNGGNRCFNNITISANGRSVVAMVVDECDSTMGCDKVHDYQPPCPN
NIVDASKAVWKALGVP
>Achn302131
MRTTYACSPAVLSQTKATLTINNFQAGGDGGGPSECDGKYHSYHLPIVLLSTG
WYNRGRRCFDNITISANGRSVVAMVVDECDSTMECDQDHNYQPPCDNSIVD
ASKSVWPSASLKITGMAWI
>Achn302141
MKRLCSKTSALLLVTIFLIACFDVEAQSCRPSGKIRGKKPPPNQCNQENDSDCC
VEGHMYTTYKCSPAVSGHTRATLTINSFEAGGDGGGPSECDVKYHSDDLPIVA
LSTGWYNGGNRCFNNITISANGRSVVAMVVDECDSTMGCDKVHDYQPPCPN
NIVDASKAVWKALGVPEDNWGDLDITWSEA
>Achn302161
MALSIGWYNRGRRCFDNITISANGRRVVAMVADECDSTMGCDKVHNYQPPC
PNNIVVTSKAVWKALGVPKENWGDLDMTWFEGIVLAPLTRQRSFDNVPQPH
AILYYSQRTTKGGLLITEATGVSDTAQGYPYTPGIWTKEQVEAWKPIVDAVHA
KGGIFFCQIWHVGRVSNQGFQPNGQAPISSTAKSLTPQIRSNGVDVAEFSPPRQ
LQTDEIPQVVDDFRLAARNAMEAGFDGVEIHGAHGYLIEQFLKDQVNDRVD
KYGGSLENRGRFALEIVEAVVNEIGADKVGIRLSPFADFMEAGDSNPKALGLY
MVESLN
>Achn302181
MRYRRNRNLELHLNSSGPTVTLATSIVYKRKAKAYCLEAKAQACRPSGRIKG
KVPPDQCKAELASDCCIEGKFYSTYTCSPPVSTPTKAVLTLGSFSNAIDGGSFS
NAIDGGSYSIGCDDKYHSDDTPVVALSTGWYNKGEKGVSISLPGGDEDNDYE
PPCGNNIVDASKEVWKALGVPRVDWGELDITWSGH
>Achn302661
MKNQGFRSAYILIICLFVTFFSSVKSQSCKPSGVIRAKKPPTGTCDIENDSLCCI
QGKLYTTYQCSPPVSQRTKAVLNLNSFEKGGDGGAPSKCDHKYHSDNTPVVA
LSTGWFNKQRRCLNNITIYGNGRSVEAMVVDECDSSMGCDSDHDYQPPCPN
NIVVASRAVWKGLAVPLSQWGELDIFWSDA
>Achn302671
MKTQVCSIVYAFIFFLLILTISSSVGAQSCKPSGKLRGKKPPPGQCNTENDSECC
EEGKLYTTFKCSPPVGSRTKAVLTLNSFEKGGDGGGPSECDNKFHADDTPVVA
LSTGWFNNQKRCLKNITIHGNGRSVEAMVVDECDSTMGCDEDHDFQPPCPN
NIVDASEAVWKALGVPEKDRGILDIFWSDA
>Achn374691
MKTFTLGASLLIIVIVLVTYCLEAEAQVCRPSGNIRGKNPPPGECNQENDSDCC

VEGKLYPIYRCSPTVSSNTKAVLTLNSFQAGGDGGGPSECDNQYHSDDTPVVA
LSTGWYNKGRRCLNDIIISANGRSVRAKVVDECDSTMGCD
>Achn374701
MKTFDLGTSLLILLILLVTHCLETEAQVCRPSGNIRGRKPPPGECNQENDSYCC
AEGKLYPIYRCSPAVSGSTKAVLTLNSFQKGGDGGGPSECDNQYHSDDTPVVA
LSTGWYNRGRRCLNDIIISANGRSVRAKVVDECDSTMGCDGDHDYQPPCNN
NIVDASKAVWDALGVPHDQWGEMDITWTDA
>Achn374711
MKTFNLGGSLLILLILLVTHCLETEAQVCRPSGNIRGRKPPPGECNQENDSDCC
AEGKLYPIYKCSPAVSCNTKAVLTLNSFQEGGDGGGPSECDSQYHSDDTPVVA
LSTGWYNKGRRCLNDIIISANGRSVRAKVVDECDSTMGCD
>Achn374721
MKTFNFGGTLLILLIVLVTHCLEAEAQVCRPSGNIRGRKPPPGECNQENDSDC
CAEGKLYPIYRCSPTVSGNTKAVLTLNSFQKGGDGGGPSECDNQYHSDDTPVV
ALSIGWYSKGRRCLNDIIISANGRSVKAKVVDECDSTMGCDGDHDYQPPCNN
NIVDASKAVWEALGVSRDNWGEMDITWSDA
>Achn374731
MKTFTLGTSLLILVIVLVTYYQEAEAQVCRPSGNIRGIKPPSGECNQENDSDCC
VEGKLYPVYRCSPTVSGNTKAVLTLNSFQAGGDGGGPSECDNQYHSDDTPVV
ALSTGWYGKGRRCLNDIIISANGKSVRAKVVDECDSTMGCDGEHDYQPPCN
NNIVDASKAVWEALGVPHEQWGELDITCFQAGGDGGGPSECDNQYHSDDTP
VVALSTGWYNKGRRCLNDIVISANGRSVRAKVVDECDSTMGCDGDHDYQPP
CNNNIVDASKAVWEALGVPHDQWGELDITWSDV
>Achn374741
MKNFNLGTSLLILVILLVTHCLGTEAQVCRPSGSIRGRNPPPGECNQENDSDCC
VEGKLYPVYRCSPTVSGNTKAVLTLNSFQAGGDGGGPSECDNHYHSDDTPVV
ALSTGWYNKGRRCLNDIIISANGRSVRAKVVDECDSTMGCDGDHDYQPPCN
NNIVDASKAVWKALGVPNDQWGELDITWSDA
>Achn374751
MLVEAYREQNRSSGLRVEGKRYPVYQCSPTVFDNTKMALTLNSFQAGGDGN
GPSECDSQWPSTILMTHQL
>Achn374761
MKTFTLGTSLLILAIVLVTYCLEAEAQVCRPSGNIRGRKPPPGKCNQENNSDC
CVEGKLYPIYRCSPTVSSNTKAVLTLNSFQAGGDGGGPSECDNQYHSDDTPVV
ALSTGWYGKGIRCLNDIVISANGRSVRAKVVDECDSTMGCDGDHDYQPPCN
NNIVDASKAVWEALGVSRDNWGEMDITWSDV
>GRMZM2G023013
MVEAKILVLVAVLALLQVSSTVARRHGKPDPCDEDDANTDDLPGGLRHKRTP
HPRSHHCAPAHHGGRGGGTPAVMTVNGFKRGESGGGPAECDGHFHGDDELIV
ALSTEWYAHGRRCHRRIRITSAHHGRTVEARVVDECDSSRGCRHNIVDSSPAV
WRALGLDTDIGQVPVTWSDA
>GRMZM2G005977
MAKTKIAVAIAILALFPLSCAAARQHGKPAHGDHDGDGTPAVMTVNGFNRGE
DGGGAASCDGRFHSDDDLIVALSSRWYAGGKRCGEAIRITANSGRTVRARVV

DECDSQGGCRNNIVDSSRAVWKALGLHTDAGEVHVTWSDA
>GRMZM2G006019
MANTKIAVAIAILLALLQVSCAAARRHGKPAHGDHDGNGTPAVMTVNGFERG
EDGGGAASCDGSFHSDDKLIVALSSRWYAGGKRCGEAIRITAESGRNREGTGR
GRVQVLDSSNLGLIKPDTLVRYRGMVQDMLGNEYYIGAFKVSENGHGNSSFS
NKPKEGDVHVSSSSTEVAGTIPEMNGGDHHIPGSSFSCLVKIYDMLGSKYFTA
GAL
>GRMZM2G317085
MAKTKIAVVIAILALLQVSCAAARRHGKPAHGDHDGDDTPAVMTVNGFKRG
EDGGGAASCDGRFHSDDDLIVALSSRWYAGGKRCGEAIRITAESGRTVRARVV
DECDSQGGCRNNIVDSSRAVWKALGLHTNVGEVHVTWSDA
>GRMZM2G397765
MAKTKIAVVIAILALLQVSCAAARRHGKPAHGDHDGDDTPAVMTVNGFKRG
KDGGGAASCDGRFHSDDDLIVALSSRWYAGGKRCGEAIRITAESGRTVRARV
VDECDSQGGCRNNIVDSSRAVWKALELHTDVGEVHVTWSDA
>GRMZM2G165149
MAKTKIAVAIAILALFPLSCAAARQHGKPAHGDHDGDGTPAVMTVNGFKRGE
DGGGAASCDGRFHSDDDLIVALSSRWYAGGKRCGKAIRITAESGRTVRARVV
DECDSHGGCRNNIVDSSRAVWKALGLHTDVGEVHVTWSDA
>GRMZM2G307222
MAKTKIAVAIAILALFQVSCAAARRHGKPAHGDHDGDGTPAVMTVNGFKRGE
DGGGAASCDGRFHSDDDLIVALSSRWYAGGKRCGKAIRITAESGRTVRARVV
DECDSHGGCRNNIVDSSRAVWKALGLHTDVGEVHVTWSDA
>GRMZM2G331599
MAKTKIAVAIAILALFQVSCAAARRHGKPAHGDHDGDGTPAVMTVNGFKRGE
DGGGAASCDGRFHSDDDLIVALSSRWYAGGKRCGKAIRITAESGRTVRARVV
DECDSHGGCRNNIVDSSRAVWKALGLHTDVGEVHVTWSDA
>GRMZM2G006658
MANTKIAVAIAILLALLQVSCAAARRHGKPAHGDHDGNGTPAVMTVNGFERG
EDGGGAASCDGSFHSDDKLIVALSSRWYAGGKRCGEAIRITAESGRNREGTGR
GRVRLPWRMPQQHRGFLPSRLEGAQARYGCRRGPRHVVRRLNTLHTELDGR
PTTEKLGTTARGSVLPIVITTLSTEKIKGPWGPPVKEIARDKNLRTTRGHPFCH
GLQDLSPLSGRSRRERETVGLQYDLVGNPLGAVRATFERTTAAAAVESGGADP
VAAFRGKDWGAGDLFRSFLLEQDDLGKVQVLDSSNLGLIKPDTLVRYRGMV
QDMLGNEYYIGAFKGGSTWRTNKYTDFSPYSMPHPCDSHIWERHLFHCVPAP
GQNSWTLESSPGPDVCRMSNCLAPELREKEERWR
>GRMZM2G429533
MATSTKLAFLAVAVLLLQAAWCGLARHHHHHGGHHDPDPCGDSSALLRHRD
HRCTSPADGGTAAVMTVNGFEKGQDGGGPAACDGHYHSNGDLITALSTRWY
AGGRRCHKPIRITSTHTGRSVVARVVDECDSRHGCKDNIVDTSKAVWDALGL
DTNVGEVPVTWSDA
>GRMZM2G343058
MANTKIAVAIAILLALLQVSCAAARRHGKPAHGDHDGNGTPAVMTVNGFERG
EDGGGAASCDGSFHSDDKLIVALSSRWYAGGKRCGEAIRITAESGRNREGTGR

GRVRLPWRMPQQHRGFLPSRLEGAQARYGCRRGPRHVVRRLNTLHTELDGR
PTTEKLGTTARGSVLPIVITTLSTEKIKGPWGPPVKEIARDKNLRTTRGHPFCH
GLQDLSPLSGRSRRERETVGLQYDLVGNPLGAVRATFERTTAAAAVESGGADP
VAAFRGKDWGAGDLFRSFLLEQDDLGKVQVLDSSNLGLIKPDTLVRYRGMV
QDMLGNEYYIGAFKGGSTWRTNKYTDFSPYSMPHPCDSHIWERHLFHCVPAP
GQNSWTLESSPGPDVCRMSNCLAPELREKEERWR
>GRMZM2G429548
MATSTKLALLSVAVAVLLLQAAWCGVARHHHHHGGHHDPDPCGDSSALLRH
RDPRCTSPAEGGTAAVMTVNGFEKGQDGGGPAAFVTDHSNGDLITALSTRWY
AGGRRCHKPIRITSTHTGRSVVARVVDECDSRHGCKDNIVDTSKAVWDALGL
DTNVGEVPVTWSDA
>GRMZM2G016741
MANTKIAVAIAILLALLQVSCAAARRHGKPAHGDHDGNGTPAVMTVNGFERG
EDGGGAASCDGSFHSDDKLIVALSSRWYAGGKRCGEAIRITAESGRNREGTGR
GRVRLPWRMPQQHRGFLPSRLEGAQARYGCRRGPRHVVRRLNTLHTELDGR
PTTEKLGTTARGSVLPIVITTLSTEKIKGPWGPPVKEIARDKNLRTTRGHLFCH
GLQDLSPLSGRCGRIDYEMFTNDLSCVLDYDRISGGNLS
>GRMZM2G334514
MATARGIAAMAMFFLVALSASHTASSLRPGAGLGTCRASGYLPGRSGNCEKS
NDPDCCEDGKMYPQYRCSPPVTASTKAVLTLNSFEKGKDGGGPSECDNAYHS
DSEKVVALSTGWFSNMARCGHRIKISANGNSVYAKVVDECDSVHGCDDEHN
FEPPCDNNIVDASPAVWNALGLDQNVGMVDITWSDTCRASGYLPGRSGNCE
KSNDPDCCEDGKMYPQYHCSPPVTASTKAVLTLNSFEKGKDGGGPSECDNAY
HSDSEKVVALSTGWFSNMARCGHRIRISANGNSVYAKVVDECDSVHGCDDE
HNFEPPCDNNIVDASPAVWDALGLDQNVGMVDITWSEE
>GRMZM2G305329
MAGVGAVAAAMFMFLLVALSAPHTASSLRPGASLGTCRASGYLPGRSGNCEK
SNDPDCCEDGKMYPQYRCSPPVTASTRAVLTLNSFEKGKDGGGPSECDNAYH
SDQEKVVALSTGWFSNMARCGHRIKISAANGNSVYAKVVDECDSVHGCDDE
HNFEPPCDNNIVDASPAVWDALGLDQNVGMVDITWSEQ
>GRMZM2G432697
MASCMKLLVLLALVQVLLSLHVQVSEATTNKHHHHHHKPEEPGSGSGSGTCR
LSGHLHGKAGNCKKLHGSDCCQEGHKYPQFRCSPPVSARTPATLTLNSFEKG
KDGGGPSECDNRYHKDSETVVALSSGWLRLDGTRRCGQTVRVTATNGRSVL
ARVVDECDSVNGCDEEHNFEPPCPNDVVDGSPAVWKALGIKESLGEVKVTWS
DV
>GRMZM2G418833
MASTTTMARISLSVSLAAILLPLALATFPHRALLQSCQSNGSIRGKSGSCNTEN
GSECCEDGKRYTTFACSPPVTARTRATLTLNSFADHGDGGGASSCTGTFFDDD
VRVVALSTGWFSRSKRCRKSIVIRASNGRSVKAMVVDECDSLHGCDTEHNFE
PPCASNIVDGSPAVWKALGLNTDDGEVPITWSDA
>GRMZM2G073114
MATVGGNRALYAVVALPLLATLLHGPMRLSHAFPYRSLLQTCQPSGSIQGRSG
NCNTENGSECCKNGRRYTTYGCSPPVTGSTRAVLTLNSFAEGGDGGGAAACT

GKFYDDSKKVVALSTGWYNGGSRCRKHIMIHAGNGNSVSALVVDECDSTVG
CDKDHNFEPPCRNNIVDGSPAVWDALGLNKDDGQAQITWSDE
>AC206980.3_FGT007
MATARGTAAMGMFFLVALSASHTASSLRPGAGLGTCRASGYLPGRSGNCEKS
NDPDCCEDGKMYPQYHCSPPVTASTKAVLTLNSFEKGKDGGGPSECDNAYHS
DSEKVVALSTGWFSNMARCGHRIRISANGNSVYAKVVDECDSVHGCDDEHN
FEPPCDNNIVDASPAVWDALGLDQNVGMVDITWSEE
>AC205713.4_FGT004
MAKTKITVAIAILALFPLSCASARQHGKPAHGDHDSDGTPAVMTVNDFKRGE
DGGGAASCDGCFPQRRRPDRGVVLAVRRREEVWRGYPHHIGQRAHREGTGR
GRV
