# Supplementary material for: The Characteristics and Expression Analysis of the Tomato KWL Gene Family Under Biotic Stress
Source: Genes (Basel). 2024 Nov 29;15(12):1555. doi: 10.3390/genes15121555 (PMC11675693; doi:10.3390/genes15121555)
Supplement: Supplementary file 1 [file genes-15-01555-s001.zip › Supplementary file S2 promoter sequences.pdf]

>Solyc01g094450.1
tttaaccttaaatagcttccaaaagggtggagatggtggtggaccatcggaatgtgataaccaataccattctgatgacactccagttgttgct
ctttcaacaggatggtatagtggaggagataggtgccttaactatatcaccataagcgctaatggaagaagtgtgaaggctaaagttgtaga
tgagtgtgactctaccatgggatgtgatgacgagcacgattatcagcctccatgccccaataacattgttgatgcctctaaagcagtgtggga
atccttgggtatacctgaaggtgattggggcgattatgacattacctggtctgatgcttagtgcatcatatattcatatatagtacttgttgtacc
attccacatcatgtatttgttacttgtaaaattacagtacaatgaaattctgttcacatcgacttttcaaatatagtaatatcaaattcattcctat
gaaagagacatgattcgtgaaatctaaaacaggacttggttcagtagctaatctaaacccactacaatttgaccacgatctatcaatcaaca
actaggccccgatctcaaatcagttgaggtcgggtatatacacctacctggattagaagctttagtagatgatgtctactattacaatatttcat
cttagtagggcagcaaaagagtaatcatacactgaaaatgaatgtgtttgaggtacaacaaattcacaaatactcaagacaatatctttggt
agcatcctatgggaagtacatggtatacaacctctttttccagttaaactaggtcttaatgatactacagagcaggcttgacgcttatatgag
agaaggtaatgagaattttcttgaagctgcatgatctttcacaaggaacaagtaagacatgagcagctgccactgcaagttgccgtagtgtgt
gtagtacttgtccaatttggaattggcaattacgttttagggcgatgtctaaatcaataacttctttagcataaggtttacctcctactactgaat
cataagcatctaaactttttatgaacataaacggtgaaatctttgagagttgcttcctgaatcttaactaaaaagtgtagcatagctcattcta
attgggattcacaacggtggaggaacagaatctgaaaaagaagtattctagcttcaaagaaacatgagagtctactagatatattttttaatt
ttctcttgttttgtcgtagcttcttctatcctagctagtggagctatgtgatgatcaaaataagctaatgatagtaatattgacgataatgtttaca
ttcatcatggacttttgcttatgtttcataccttgagtttcattgtgagctatcaaggtcatttgccatttttaaccagccttactaacacttcaagtt
tactttataaatgcggaattagagtctatttcccctctcctacatttgatgaggttaagttggcatgtcttgcctcatcttaatttttatatttttgct
cattaataaactaagtcatcatctagttgttttaagattaaaagaatattggtgctgctactgaacaacaaaaatacaaaattgtattcagcaa
ctaaagaatccaagacaataacatactccaagtcaagttcttgccatattatgttgctgtagaatgcctttctacaacaggatacagaaaattc
taaattagttatccgctgatagaaaccttcaaaaggtaaataaactttaatcaagtatgtccaaatttactatagcaaaatactaattaaaag
gataaagaagcattccaacagacaatattgactgctaaaacttaataattgtatattttattagaagaaacttttcttgctttgagaaaaatatt
ggccatttgtgtgaacaaagcactatataaaccagttagattatcccatctccacacaaccaacattttaaaacaaaaaccacaaaagaaaa
cagtaagaaaaatc
>Solyc01g010400.1
tttgtcatatgatgtgtgtatatcctttaatatctcttcttctgccactatttgcgacggacctacatcaaagctatcttgcttaaagccatgaaat
gcttcgttaatcaataattctataggatttttggaggcagtgtatcctgagtaaactctctgcttttaaaattttggaacacacttgtttccccat
gcataacgcaaatgacatcattttcaggaaatgacttgcaaatcaaatgatcaaatactacctctctagtctgccattttccaaaacaacattg
aggacacgggcattttattctatcttctatagctccatttttaaaagcaaattccaaaaactgattcaaaccaaccagatattcctttgtggtcct
tggcattttaatccaagatttatccatcgaaaaaataaaattagcagacaatatcacctataagattttcatgagaaaatagtaaggttagagt
gatatttaacaattttatgtaccagctaatgctgaaatggagtatatgcagaaatggtcataatgtattctttttgcatgccaaaattgtaagtg
cacctgaattatttacaggcttgtactcgatattcaatcaatttgtgtatgtactaacttattcggcagaaattcattgaattattttgtaagtaaa
ataaaacagatagctgagattgagaaagatcagcatcagcatcaacaaatgaacttgataccaggaagttcatcaagctatcatcaaagaa
taccaacatatatcaaaagggtgttagagtacctcaactttctttatccttaaacaaacaagatctagcagctctccagttccagctccagttca
agataaagaaaatattgaaagggaggtcaaacctgataaattaaacaaaagaaatatcttctataagattatgcaaaaggaatatctaaac
aacagtcatgatatcacaacatttgcttatatagaacgactaacttaagttatgaatctatctatgaaaactcattttatgaactagccagatca
ctagaaatcatgtcactcaagtcagtattcatcaaaaaatggtaaaatatgttcctgaatcctcttactctctagtttaatcagttagttcctctgt
cacggatatgaccatggttagtgaaacagagtaatttgtcatatcatagtatgtttttaggagtcttatccaaattgttgatgaacatacaaaca
ttatgtgatgaaaacaaaaatacagataatactaacatcaccaagtctaattcaagtttatcatatgtcaacttcaagatcaatgaagaacta
attcaatcttagatcaaatcttcatcaataaaaagtctgtttaagattttgattatttattagtaatttttaaaaagaatttctacttatttatataa
ttgaaaagatgattgctagaaatccaggtgttcatgtttagcgaccagatgaaacaagtctgttcttctcacctaaatacctacggaaagatac
aaacttgtattcaccactccaatctagataactactgattatagaaaaaatggcatgatcgtcaaggaaagtacaatatagtatgtgatatgt
ccactccagtacaacaaaatcacaattaggagtaatctatttgactgctaaactcaatagttgtatgctttgttaagagaaaacttccttgcatt
gagcagaatattggcatattgtctgaccagaaatactatataaactagtttgatcatcgcaatctccacacaaataacgtttcaaaacaacac
ctaactagaaaacgggaacaaaaaccatgaagtctactttaggaacatcacttttggttttgctggtttttgtcactcttacttactgcttagatg

caagactacaggcttgccagtcgagtggcaaaatcagagacataaatccactccaggacaatgtaaccccaaaatcgactcagattgttgc
aaaaaaggcaag
>Solyc01g094430.1
aacgactttctgatgaacagtcagagttgaagagtaatattcgagaggtatgggcagttgcagatctctgtatatgtctagtttaacatttcagt
ttatctaaacacaaacagttgtttcattttatattttttgggctttgttaggtcttcaaagcaactgagttgctcaagaaacaaatgatggtgtca
tttgcagatgtctttaaagcgatcgagtcattgtcgaagaaacaatcagaaaaggataattcagaagcattgcatgtaaaattcccggcacta
ttttttttttgcagtttctgcatattattgtaatttatgttccattgttcgacagattgatggaagagatggccaccttgacagacacggtgatggt
gattccaatggttttgacgctaacaatggccatggtagtgatggcccagattgtggcaaggactcccttggtgacaaggagaatagcgagaa
agtcaatattggagccttgagtggaattacaaacacatagggaaaatcatttttttccctgtcctacattttatgaggttagaatttcctgactaat
tttttgtttaataaatgaagtagtcatcttgttgctttaaaattttttgtactctaatggtttggtttgggacttacttaatggaagtctcaagtttga
aacctcttacctgcgacagcagggagttgaccttatgggtctcgtatcatggggcttgcctagtgcgaatatctctccggtgtagtttgtgagct
attgcacatgagcggggttaaccatatgtgcaccagtttgcaagctattgcatgggagccgggtttccctatgtgcactcaaagggtggcaat
tgtgggttcccttgtcataaaaaatggaagaatacaatattatttccttgagtggttgctctcatgttcctctattcacttcaattgtcataacttat
attcagccgtaggaattaaattgatttttcatagcactcctgtccatatcaaaagtcaagccaaaaattctgattgtaccttgaatttgtggccat
ttttcttagtaaggtttgtcttcacgacaccagatcagtttggtgatgatttgactgaaggtaataacaggtactccaggttgcagaaactgttg
cagggttgacaaagcaagatagagtaggcaaagcaagctgttgtatgttcgagtgtctgtctagtttcggattttcacaatacagttcaagatg
aatttggaatcagcaaggaggatgaggtcttcttccctactcaatccacaagtgttgactatttcaactgggtggtgtaatagacatgtactca
atgagagtgacaaccgcggggtgctgattggcctccatgaccagctaatgtggttgctgcctcaaagctgtctgtttcctttcgtcaagtttgtt
ataacttggtctaagaacttgcttatgtaatttatagtccccatcatttcgatcacaacagcatttctggagtgtatatgtgaaatttattggattc
aattctatgcaatcataatattttcatagtaactagggatgaatataagctcttcgacactgtaatttctacttatttatataattgaaaagatga
ttgctagaaatccaggtgttcatgtttagcgaccagatgaaacaagtctgttcttctcatctaaatatctacggaaagatacaaacttgtattca
ccactccaatctagatatctactgattatagaaaaaatggcgtgatcgtcaaggaaagtacaatatagtatgtgatatgtccattcaatcaca
agtaggagtaatctatttgactgctaaactcaatagttgtattccttgttaaaagaaaacttccttgcattgagcagaatattggcattttgtctg
accagaaatactatataaaccagtttgatcatcgcaatctccacacaaataacatttcaaaacaacacctaactagaaaataggaacaaaa
acc
>Solyc01g094440.1
aatcacaagtaggagtaatctatttgactgctaaactcaatagttgtattccttgttaaaagaaaacttccttgcattgagcagaatattggcat
tttgtctgaccagaaatactatataaaccagtttgatcatcgcaatctccacacaaataacatttcaaaacaacacctaactagaaaatagga
acaaaaaccatgaagtctactgtaggaacatcacttttggttttgctggtttttgccactcttacttactgcttagatgcaagactacaggcttgc
cagccgagtggcaaaatcagaggcataaagccacctccaggacaatgtaacaccgaaaacgactcagattgttacaaaaaaggcaagat
gtacaccacttacaaatgttcacctccagtgactggtaataccaaagctgttttaaccctgaatagcttccaaaagggtggagatggcggtgg
accatcagaatgtgataaccaataccactctgatgacactccagttgttgctctttcaacaggatggtacagcggaggagatagatgtctcaa
ctatatcaccataagtgctaatggaagaagtgtgaaagctaaagttgtagatgagtgtgattctaccatgggatgtgatgacgaacacgatta
tcagcctccatgctccaataacattgttgatgcctctaaagcagtgtgggaagccctgggtatacctacagatgattggggagattatgacatt
acatggtctgatgcttagtgcatcatatatagtacttatggtatctgtttgcatataatggttgtctttgtactcagattatgtcatctagtttctcat
gtgatttgttgttgtaaaattacagtataattaatgaaattctgttcatgactacttttgaactgatcataatatgaaattcatgccaatgaaaga
gaagagacattctctctatcagtgaaagctaaaacaggacttggctgtgtagctaaactaacctactacagttaataaaaaaaacatttgatg
atttaaagttaaaaataaggtggaattcatcagtcccaaatgcagtatgttttaactctaggagttgtatttctttgtgttatgtttggtagttagt
tttaactgtaatcattgattttgataacaacaatgaaaataagctcattgaacaatataacgtactaatacttcatgtgttcatattagtaccctt
gaaacctccttataaacctatggttccaactagtacactaatccatttatgagagaagttgcaaaattagtcaggtggaaaatcataaaacag
atcaaaaacgatttctgatagttcatgaactatcgaaatttaatgaattgaaggcgtaattcttatgttactgcagcaaatgttctcttccaaca
ggcaaatggagcagtgtggatggagaacattagttgatcattctcacctttaaattagtcttagtctcctatatcgaccgactatcataactgat
gtcacatgggaggatgtgacaacatacacagtttaatcccacaagtggggtcggaaaaggatgatcataacaaaatgaagactttagtcat
acagatgctagaatacttcatagtaacttgatgaaacaatctgttgttccatgtctatagaaaaatacaaaactgttttcagaaacaagagaa

tccaagacaataaactaatccatcaagttgacacacaaaaatctaatttacacatctatgtagagaaaacaaatgcttttaccatcaaggga
agtaaagcaattattgtcacaaattataaaaatacactcattaatccatttgactgctaaacttcataacatatatatacttaataataagaca
attgtcatgccttgaacagaaaattggcattttgtgtgaccaatacactatataaaccagcttgatcatctcagtatccacaaccaacattaca
acaacagaatcacaagaacaagaacc
>Solyc03g013300.1
ttatcttgcctaaagccatgaaattcttcgttaatcaataattctacagtatttttttggaggcagtgtatcctgagtaaactctctgcttttagaatt
ttgtgacacacttgtttccccatacataacccaaatgacataattttgaggaaatagcttgcaaatcaaataatcaaatactacctctctagtct
gccattttccaaaacaacattgaggacactgacattttgttctatcttctatagctccattttaaaagcaaattccaaaaactgattcaaaccaa
ccagatattcctttgtggtccttggcattctaatccaagatttatccatcaaaaaaaaaattagcagacaatatcacctataagattttcatgaga
aaataataagtttagagtgatatttaacaattttatgtaccagctaatgcagaaatggagtatatgcagaaatggtcataatgttttctttttgc
atgccaaaattgtaacataatgaacacaggaagtggatgtggatttactaaacaacaatctgagagaaaatgtctattttttctctatttagta
ctactgtagtaagcgcacctgaattatttataggcttgtactcgatattcaatcaatttgtgtatgtactaacttattcggtagaaactcattgata
tattttgtaagtaaaatataacagatagctgagattgagaaagatcagcatcagcatcaacaaatgaacttgataccaggaagttcatcaag
ctatcatcaaagagtaccaacatatatcaaaagggggtcagagtacctcaaccttctttatccttacacaaacaagatatagtagatgtccag
ttccagctcctgtttaagataaagcaaatattgaaagggaggtcagacctgataaataaaacaaaagaaatttttttctatgagattatgcaaa
agaacaacagtcatgatatcacaacatttgcttatatagaacgactaacttaagttatgaatctatatatgaaaactcaagttatgaactagcc
agatcactagaaatcatgtcactcaagtcagtattcatcaaaaatggtaaaatttgttcctgaatcctcttactctctagtttaatcagttagttc
ctctgtctcggatatgaccatggttagtgaaatagagtaatttgtcatatcatagtaagtatttaggattcttatccaaattgttgtcatatcatag
taagtatttaggagtcttatccaaattgtattctttattagctttaatgcaaagagcaacgtcaaaccagattgaacgttcagaatgtcatgtat
attacccaagactctgaatcctaaatgttgaatctgtcttaaaatgataaactagaatgaacctaaacagaagaaagactgattaatgagaa
catatttaaatcgattaagcaaggacgcagtttttctgattcctcatgagttataaaggtataaatgtctctgacgagatgttcaaagatacaca
caaactaccagaagcttgatctattttggctgaaaagggaaatgtaaatgagtacaaattatgagataaacaaataaatgtcaactgcaaa
ccaaaagctttgttaactccattaatatgatagctgcaaactaaaaagtgtactacagaatattctatccctttgcatgatctatgccacattaa
gagaacttctcttcattctataactgcatcgatatgtaacaaatcctatctatgcccattaaaataacaaagtctcaaaaatttacagcatttct
acagccaaatcacaatcaggataatctatttgactgctaaactcaatagttgtatgatttattaaaagaaaacttccttgcattgagcagaata
ttggcatattgtctgaccagaaatactatataaaccagtttgatcatcacaatctccacacaaatagcatttcaaaacaacacctaactagaa
aacaggaacaaaaacc
>Solyc05g042020.1
ttatgtaccagctaatgcagaaatggagtatatgtagaaatggtcataatgttttctttttgcatgccaaaattgtaacataatgaacatagga
agtggatgtggatttacagaacaacaatctgagagaatgtctattttttctctatttagtactattgtagtaagcgcacctaaattatttacaggc
ttgtacttgatattcaattactttgtgtatgtactaacttattcggcagaaactcattgatttattttgtaagtaaaataaaacagatagctgagat
tgagaaagatcaacatacacatcaacaaatgaacttgatactaggaagttcatcaagctatcatcaaagagtaccaacatatatcaaaagg
gggtcagagtacctcaactttctttatccttaaacaaacaagatctagcagctctccagttccaactccagttcaagataaagcaaatattgaa
agggaggtcagacctgataaattaaacaaaacaaaaattttctataagattatgcaaaaggaatatctaaataacagtcatgatatcacaac
atttgcttatatagaatgactaacttaagttatgaatctatctatgaaaactcaagttatgaactagccagatcactagaaatcatgtcactcaa
gtcagtattcatcaaaaaatggtaaaatatgttcctgaatcctcttactctctagtttaatcagttagttcctctgtctcggatatgaccatggtta
gtgaaacagagtaatttgtcatattatagcaagtatttaggagtcttatccaaattgttgatgaacatacaaacattatgtgatgaaaacaaaa
atacagatagtactaacatcacaaagtctaattcaagtttatcatatgtcaatcaagatcaatgaagaactgattcaatcttagatcaaatctt
cattaataaaaagtctgtttaaggttttgattatttattagtaatttgttgaaaagaatttctacttatttatataattgaaaagatgattgctaga
aattcaggtcttcatgtttagcgaccagttgaaacaagtctgttcttctcacctaaatatttacggaaagatacaaacttgtattcaccactcca
atctagataactactgattatagaaaaaatggcgtgatcgtcaaggaaagtacaatataatatgtgatatgtccattccagtacagccaaatc
acaattaggagtaatctatttgactgctaaactcaatagttgtatgctttgttaaaagaaaacttccttgcattgagcagaatattggcatactg
tctgaccagaaatactagataaaccagtttgatcatcgcaatatcgacacaaataacgtttcaaaacaacacctaactcgaaaataggaaca
aaaaccatgaagtctactgtaggaacatcacttttggttttgctagtttttgccactcttacgtactgctttgatgcaagactataggcttgccag

ccgagtggcaaaatcagaggcataaagccacctccaggacaatgtaacaccgaaaacgactcagattgttgcaaaaaaggcaagatgtac
accacttacaaatgttcacctccagtgactggtaataccaaagttgttttaaccttgaatagcttccaaaagggtggagatggcggtccactcc
agtccatttcttagatttttttctgagtcagaatgttcaaaacatcatgacacctattttattgttgattcctcctcttttgatggtattatgtgccta
caattttaaagttttactatagaaagtcgtacactttttaatatcgaggaaaagtaaattcatatttgattgcatttctactgtgtttcaagtgggt
cgatacatgttttaagaagacgatttatccgtagttgtcttcatatcaaacctattttccatcttcatcatttttttgatacttacacccatttgctcaa
gatcacagg
>Solyc05g048780.1
tctgttctttaacatactatttttgactgctgctgctgctgcaaaatgtcactgcatgagttatcattcaacatcaacttcaaccatcaatgttgtt
atcgttgtgatcttctacaggtttcagtgcttaggagtaaaaaacagcaaaataaagctggaattgatgagtttgctgcacggattaatctcgt
ctccaaggaacagtagtgttgcagcaggagttgtcaattcgacaaaagacttgccatttcaatggcatccatcatcagttgaactaatcatcc
aagatacgtctgagaccaatagacttgcagttgaaggaacgtagttgagccaaaagctaagagaggaataactcaagtttttatttcatatg
gaaaagggtctccttttgggatgaagagttaatgaggtttgcctcatttacacaaaaagctaaaaaaatacttgatttgacatttgtaatctgg
ttgtgttgttgcaaagaagagagaaaatttgccaaataatgggcaatggttttgacaggagaaagaaatgtttatttaacttagttgtccatttt
tgaaaagtggaaaaaaatgttgaattttaagtaataattattgaaagctgaaggacacaatcataaattatttagcttacacataggcccctg
gatatagaagtcaaagttctacccaagaaatgaaccttagtttttcttcttctatcttagatatagaggtgtttatggttcaatttgggtaaaatg
ataaaatcaatttgtatgaagagatctctgtctatacctacctcattgtatatattgacagaaaccaaaacttaaaactattgagtctaaatgtg
tactatctgtgctgcttataaaattctctttttaaaattcaaagttatggtcttgccacaattcccttgcattgggttatttataaaaggttaaaac
tagaattgtaggggctctcaagaaatgagaactcctccaaaaattggtgtatgccactaaatgcaatgcacacacggtattctttagtaaaag
gcgaaagaatagttcactttgtgctcactgcgtgggtttagcttctgtattagtgatgcattttatattgtacattaagtttggattcaccttctatt
accaatgtggtactttacctatgaaagggaagaataataaaaaataataatttcaaatctaacacaagttataaaatattaatactaattttta
ggggttaagattatgttgtacattttgaattttgcaaagaccgcaacgacatacaactacataatcaatgtaatctacaaaaaacaaaaaaa
actttatgtgtaacagtaaaattaataaagagtgaatgcgataaaatcgatatctccgtcattttgttcaagattataaattctcttgtgctgccg
ttacaaagattcttatatgcatgactttttttcatgacaaaaggacaacaaaatctagtctccatcaagttaacaaagatgccccaaggcatttg
tcttgtgctctcatccgcggacttctcgtggacagtcaacgttgacatgaaacataaattttagagatgtctagatgattatttggtaagctaga
gtgttcatcgacttacacaataaagacgagttaacgtgtatagatgtgcatatacgtaatcaatcagagtgtttacttactagagaacaaattt
gagtgccttgtttatgtcatatagccaaaaataaccccaacacaaattcaactgttgttgttaccttgttagataaaaaccaagcatcttcaata
aaacaagttaaaaattctcttgggagatcacgaattaaccattaataatttatccatacaccaatatttgtcacactataaatagttctattttttt
aaccctttcttcaccccttcaccaacataacttataccactacacagaacgtctaacgatcttataataacactcaccaaacacaactcaaac
aaagacgtaaaaaa
>Solyc05g048790.1
aattctaattttttaaaaaattctataataacattaaaaaaaaacatacaaatatatatatatatatatatatatatatatatatatatatatat
aaatgtatatatatacggatatttcatgtttaaaattaatggacgcataaaaaaataaatttaaaaccatttaaaataaataagtcaatttcaa
ataagtttaaaataaatttaagggtaaataaaaattttattattaaataaataatataatgtccaaaaattaagttaagtcattctaaagttaat
aaagcgactgtgctagaaccacgggactcgaggggtgcctaacaccttcccatcggtcaacaaaattccttacccggatttctagttcgcaga
ccaataaaaatagagtcaaatttccttttgattagggatttaaataaggtgatttagaacactaaaactcaatttcaagtgacgactctaaata
ataattatccctttccaaaacgtcactttaattggaaaaactctttttctttcgaaaaaataaaaatcaaaaaaaatattgagagaaaaaaaa
agggggcgtgacactaataactgctacagaagaaatagatgcattgaatcatcaatcttgaatactatatatctatggttcattttcatttccaa
ctctaaaaaattcagcttgcattcatccctcattgctatcgttgacgctgaattcgcctatgaaaatgccacaaaattagagatgaaaataaaa
tttctcatgttattgaatgaacatatttacctccctactcaagttcaacatgcaccggaaatgtacctcgtattcaacagtgtggcctagtgatca
aggaggagaaacaacaatcataaagtctcaagtttaaattccaatagagattaaaaaaaaaatactaagtaaattcttgctatctacgcaagc
ctcggcagaactaatacaacaagagaaattgttttaggtcacaaagtttgaattcctactttgatattttagtatcttttgtgaatctggtcctgtt
ccttcactatttccccccgctaaatgtgctcgtgagagatagtaggtatcaaaaaaaaatagttaatgttgcgcaagttggcctaatatcatattc
attaaaacataaaaaagactctaaacgtaactctcacacaactaattttcttaaaaaggataaatgatggaataagccaaattcaattgaag
ttttgctaagtgcctaagaaatgttcaaagggctgagtgcttttttttttaaaaaaaaaattattcataaaataatttgagataattattcataatta

tgccctcgtctcacatcttcatcttctttcatatataagtgcagttgaatttataacgtgatttaaaggacatgtggattgaattaaattgaattca
aattcaaaatttttttgttacattaaatatgcttatttggtggaaattttaatagcgtggtgtcttagttaagctgattgagactatctttctttcca
aatttgtttttgaatcaaattttgttgtcatgaaaaaaaattcatatgccaataggaatctttgtacggctacaagacaaaacaatgttatattg
atgaaaacatcaattttctaagggtcattttgtttaacatatactccctcccgtttcttcttaattatcatatcgcgcttttaaagaatcaatttgac
taactgtcaaagctaaattagattatattataaaaatttgaatatttaaaaactataaaagaaagtactagaagttatggttttagtcaagtca
aatgataatgaaaaaaaaaattgtgacaagtaaaagggaacgctgagagtatatatacaacaagcttagggaataaattggatatttgaaca
caaggttggagttttatgtaatcaaactttatatagactaataaaccatctcccaaatacacaatcaaaaacaaaacattttctaattaagag
aaaatttgatc
>Solyc08g080950.1
acgacttgtgtttatcaaaggtgggaccaaggtctgtgtacctcctaccctccctaggtcccacttgtgggaccacactaggtatttttgttgtc
gtcttgtgtgtatcagacttgtctcttggaaaccgaaaaaagaacttgttatgtgttcaatgatctatgcataatgatccgttggtatgccttaga
agaacacaattagaatctgtgctgcatcaaattccacttaaaatgatagtataagatccattatccatatagtcctttcaagattgtttatttctc
tttgttacaatgtggttaaagttctacttacaggaacaagaactatgatttctagatcggagcgaaacatttttatttttaatggagaactaaa
aaaacatggatatgacgatgagatataaacatttgatgctgcaaatataacaattttagcaacttgttcaacatattagtcaatataataatca
tataccaaggcttgttaatttaatggagtacttgtatactaaatttattaccttctcaaagaaataaaactaatggactacccatgactcgaga
accttaacctccaataaattgtttgatttttactaattaacatgtacatattatagcattccccaattaagggtaacttgcctcgccaattctcttttt
cattatagtaaaaaagtttccacatgtatcatgagaagtacatgtttcccattctatgcagggccgtttcgagatactatctctgtctggttctta
cttggttgctgaaactggtggcccacgcactcgcactggtggtataagtatttcagtttgtagtcctgatggccatgtaattggaggtgctatag
gggtagacttatagcagccagccctgttcaggtactctggcttgtagatcagatatttgtcttctgttttctggtgtaacaagcaaatgaataat
atttgccacaggtagtcgtatgcagctttgtctatgatccaaagggaaagagcaaaccagaaagtagcactagagacgagcaggagtctgc
tgaaaagtcatctacaccagggtctggtcggagtgtctggcctccaagttcccgagcagatgtgagaaattcccagaccgagattgatcttac
gcgtggatgacacaattcgctggaatgcatatttgaggagtttgtatatatgtgctattgtaaatgaaacgcgtcaggtgatcaagctcaacga
cttaactgtctgccaattagtagatgaaggattatgctaactttggtaacaaaattctccttgtaggtactgtttagtggtaagaatgatatggtt
gttacaagtaaaaattattgtttccttagaagtgcttttggtctcttctgttgtaacttggaatatttttatttttgggacaaatcaacaggccaa
acatgagttcccccctttttgcctccatgttctgaagctaattctctttttattttttggtagaatggactgaatctcagtggatcgtgtcagactact
agtaatgctagtctgtcacttgcacatcgatgatatcacccccctttgtaataacaacaatatactgatgtagttccacaagtaaagggtacaaa
aacagcacacgcagatctttattccaatctcgtgaaggtagaaagattattttagaaaaactaatctcttcagataagagtatttaaacaagta
aaattttaaaaaaaatgtcacatccacaacacaaataacagagttgcaatttattttacacatacaacactgaaaataagttttattatctaat
aaaaccaaaactaccctagtctattttaattcctctccacattgttatttttcttgtgatgttactttattacttttactccgttatgttcaatggtttg
gataaacattaccaactctacttagatggcaaattcttctataaatagcaaaccaattctatcaactcaaaacatcaagaaaaattgaaaaa
aac
>Solyc08g150140.1
tatagaccactgatctagaatgtttctgatttgtttacaatctagggaaagcaaattaacaacaaattatttttaaaaaaaaaaaactgctcagc
tcaactttagtgataaagggtgcttaaattttcttagaatcgattacaaaggtgaccgaattaatatagcggatgataggctatagatcgatta
caaacatacacttaaccacgattattttaacgagggacaagccaaaaaaatatgttaatatagtactcaaaatgatgaaattaatgccctttc
agaaacgataacaaaattcaattttatttgtttcatgtaagcaaaattagtgtgaacttttagaaataaactcaggaaaacactttcattacaa
atcttatatttgatagttttcaagaatgaaccatgtaattagtacgacctcctccgttaagaagtaaccaataatactaaactctcggtcataaa
aaatcaaaatattcataacgcttgtgatttgttccacaccctaatttaatcaaaatacttagcatcaagtacttttctatcccttgatataattgcc
tgttaatatcccttggtataattgattccattagaaattcatttttaataattatagttatttcaaaaaataattaaaactgattcgtccgtcttat
atttatcaaattaaaggtaatacgaggtttggtatgattcatcctgtctataaaaagatcaaataatttcatgaagaagaacaaatatcttggc
ctctaattttgtaaatatgaagatattattaaagtttttgatattattaaagtttttgaagaaaaaaaaaaaaggaaaaacaacaatgaaagaa
agatggactactttttaagagtaaaaatattttaggaatataaattaaataaattaaaattttatattgataatcaaacttcctcacaacaatat
ctaagataaatatataaacaacaaatggctagtacgttatttattaccttaaatcgatacttgtcaatataaataactaataatatagatatatc
atcaatatcgatatcttgaattaccaaattaatttttttcttttagattaattgaaataaaatttaagatcagtgattttttttatcaattagaactat

aattaaataaaatttaaacattgttgactccttatattatttatttatggcatgtaatgtgagtttttcttagttcctgttattaatggacttctcgaa
ttcccccatcatgttagtatgtttcatttttttatttgtgtttgaagttccatttttgtttatccataataaattttttattcgcttgttatagtgaaat
aatttagaatttattaatcaatgatcttgagttagcagaaaattttcttaattttatttttcttctactgttcttttcaaatactatgactatttaaatt
attattttttcgtttgcattttattggaatagtcaattttaattcgatgacaaatgtttctaccttcttgcttaagattcaccatccttttaattttgtct
tgtaaaccatatcaaggatatacgtgtaatgattattattattgttttgaaaaagattaattagcctcgtgaaaaccttgaatgtagccaactttt
aatatatagtaaattaacatcacaatcaattcttttcaaaaaaaaattactgtacatgtttaaagcaacttaaaatttcaagaatgtcatctag
aaaaaaacaaataaacaatggcgttgcaacatattttaattatatcatgatttggtttgtctaataaaatccaaaacattaatttcctagtaaa
acttgatattgttcttgtgtcattcatttttttgtcagtattcttacccccattatgtttttaattgatctatttatatttcacatatttattggccggca
aatctttctataaaattatcaacttttcaccaatttaataaaattctcaaacttacacctaagtgcaaaaa
>Solyc11g069870.1
caaataagtgtagtagttgttaaggaaaacattatcaaatgaatttgatactttttttcttgaattacattatattttttgtttctgcctttttttattttt
actaaagcctgcacttaaattgttctttgtgcttaaggcccaatagatctgaagcacttttttataactttttgcctttgactatactgctttgcagc
aataactatttgaactcaatgtagaaggtgaagagaattgttttacaacaatagattatgtctttgatcagaacttttatcaaaactctgcttttt
cctcttgttagatgatggatggaacatccaggatgccattagtgaaatcgttggcagacgcaccgtgccacaagttttcatcaacggtaaaca
catcggaggatcagatggtaagctaactcaaagcatatagctgcctctgtattagtcatagtcctgtgcttacaatattatttactttacagata
ccatcgaggcgtatgaaaacggggatctagctaaacttcttggtgttaattctaagaatgatgatctttaagctgaatttgttacatggtttcag
attaaatgactctgtaatatgatatatgatcaccataggtctttttgcagcctttgagggtgtctgtgtggagtttgaaaaagttggtgattgtaa
gaatgagcctttacttcataagaactttaggataaaggacttgttattgtaggtataattataattatgctatcattgctattactccgttcattta
aatttgtttgcctggttttgaattggcatgtagtttacgaaagtaaaaaggacatctgaatattatggttcacgaagaaagttggcaaagaaa
ggaaatacgaacaaattgaaatggagggaggattttcttgttctaatattttcatcatacctttatattcttgcattctttcaaactatgctttgga
aagtcaatgatctattcgaaataattttttaatcttccaaaggtagggataaggtctgtgtattaattacttgtccttccttgattgcgctttttag
attatattgattatgtttttcttgttgatttatgtttctaccgtacaaagtttgtattacaaaccttgctagcgattattcatatattttagatcaaatt
gtgaaaatttatcatcaagtatttatttggtcgtgagattcgatcagattttgaaaatcaatcttcagtgttcactcagaaattttaattttttttcaa
gtaaaatgtatgttcaaaacacataattttaatatattttctatttgaatacatgatctagacaaaagtaactgaaatcggaattacaaatattt
gagcaattattgaagtcgttgcaacaacttcaattttatattccagcaacttgtgaattcaatacaatgtatcaaaattgatcaggatcacctaa
gataatattttcgatattctatgatatgaaatcttattatatagtttcaaatattaaagtatttacttaataattaacacgtttttcaaagcaaacc
atgaaaattgaagttgattgagccaatagataaccatataatataatatagtaccagatataatgatgtactcgatcagctataaaagatatt
gtatatgtatggatacattatcatgtgcaagcataaaatatatttcacttttattatacgagcgaatacaatgtatttcactatattttatgtttact
ttttacatatcgttatttagcaaaatttaagtgtaaataacactatttactataaaaaaaattcaaaaattaaaatttttgactccgtcgcctata
ttttggcaaaatgaaaattgtacatcactagtcgaaaatcacgactaaaaaataaatcaagtaaatatggcctacttattaattattgcctata
tatatctacctattttgcaatatctttattactcatataaccacattctcgatcaattcttacaaaaatagccact
>Solyc11g069880.1
tatacactatgtgataagatagacatgattagtgttcttcaagaatttgctatgaaatatttctatataattctactatttcattccctcaattctaa
ttcatttggcttgattcagagtttgagggtaaaattaataatgttttgtgtaaattcagatatataatcttcagttatatacttagaaacaacaca
ctatatataatattgaagcctaaagggtgaaaaacattaataaagtttttaaagaaaatcgcttcaaagacagcaatttaccttttttttaaaa
aaaattttcaagcaattagacatgtatgtatataataaaataatatgtaaagagagttataactttttgagaggtgaattaaatggaccaatat
ttaaccattcaaaatatttataaactttttgaaaaaattatgttcaaacaattatttttattagacagtaactatgataaataaattgaaacgaat
tgaataccacatgcagctacaaataagcaaaggaatatatgtccatttcaatttttaaatatagtttataaataaattaaaacaaatgcctaac
aatattcgactttcttaattaatcagataaacatatttgaaatcaaactatgaaaattgacaattgaatttacacaggacttttctagaatgtgg
actatcattttatgtttttattttaattagaagaattaacattaatagtcttcatcaatataattcaaaagatttataattaattttagcgtcataag
gaattagctgtcatactcctatcacaaatatctctattataattatgctagatggctaaaatactcgatattttaaaaatgaaaaattcaattcat
tttatttatttcgaatttatctttttctcttttcattttttttaaaagaaaaatatctaaaattatatttatttaaaatatccattaattgaataaattatg
aatgtattgtaattaattagcagccactacctagggaaagagtaacagcaatagcaaatctcactatggtctaatttatttttttcccttattactc
ccctacttttttatttcttcatttctcatatgttaaattaaaatttaatgaattaaaatattctcaattaattaattaatcatttatacaaaagttatta

cttgctcactatatttatggtataatgacgagctcgtaagtaaccattagctttttatgtttggatgaattttagttgatgaaatctttttctagcttc
ttttgatatttttattaaaattcgattaaatttaaatttgtctataatgatcgcgatccatttattaactagtagatcaacttgttagtccatctatta
atcctattgtgcatccatgccaagtctttgcattgaatagctagaaaagtctattttttttagctaaattaccgcaatgaatataatattaaatttc
gagtcagattgcacacctaatttaaataaatgaaatatctctcacttctcatcaacaaaatatatatatatatatatatatatatatatatatata
tatatatatatatttatatgtatatatcaaataatttttctgccaaaattaaaataaataaaaagaaattatgttataatgatcgtgtatatat
atatatatgagataattttttttcaccaaaattagaataatgaaaagaaattactttataataatcgtctattactctattttattgactaatttttt
atgagaactttgaatcaaactattattcattgttagttcattcaactcaattaatttaattttgatacaatttaataaataacatatttaggcttta
ggtcaatcatttgacatgtgtacaactctactagccgaaaattacgacttacaatatcattttttcctatatatattcatacattaagacagtttt
attcaccaatcaacactattcttacacatataaccctttcaatcattacaaaaatagccaca
